# Unveiling the genetic diversity and ancestry of *Brassica rapa* weeds in Argentina: evidence for local adaptation and feralization

**DOI:** 10.1093/aobpla/plag018

**Published:** 2026-04-06

**Authors:** Sofía G Tillería, Alejandro Presotto, Claudio Pandolfo, Alex C McAlvay, Eve Emshwiller, Makenzie E Mabry, Kevin A Bird, María Soledad Ureta

**Affiliations:** Departamento de Agronomía, CERZOS, Universidad Nacional del Sur (UNS)—CONICET, San Andrés 800, Bahía Blanca 8000, Buenos Aires, Argentina; Departamento de Agronomía, CERZOS, Universidad Nacional del Sur (UNS)—CONICET, San Andrés 800, Bahía Blanca 8000, Buenos Aires, Argentina; Departamento de Agronomía, CERZOS, Universidad Nacional del Sur (UNS)—CONICET, San Andrés 800, Bahía Blanca 8000, Buenos Aires, Argentina; Institute of Economic Botany, New York Botanical Garden, The Bronx, New York, NY 10458, United States; Department of Botany, University of Wisconsin-Madison, 132 Birge Hall, 430 Lincoln Drive, Madison, WI 53706, United States; Florida Museum of Natural History, Gainesville, FL 32611, United States; Department of Trait Diversity and Function, Royal Botanic Gardens, Kew, Richmond, London TW9 3AE, United Kingdom; Departamento de Agronomía, CERZOS, Universidad Nacional del Sur (UNS)—CONICET, San Andrés 800, Bahía Blanca 8000, Buenos Aires, Argentina

**Keywords:** *Brassica rapa*, genotyping-by-sequencing, herbicide resistance, feral populations, population genetics

## Abstract

Feralization is an evolutionary process that can reverse domestication traits, giving rise to invasive or weedy plants. *Brassica rapa* is an example of extensive diversification, producing multiple domesticated crops, while its non-crop forms persist as weeds in disturbed habitats worldwide. In Argentina, the spread of this weed has increased since 2012, when populations resistant to glyphosate (transgenic) and to acetohydroxyacid synthase (AHAS)-inhibiting herbicides (non-transgenic) were first reported. Despite their spread across more than one million hectares with economic consequences, their origin and genetic diversity remain unknown. This study aims to evaluate the genetic diversity and population structure of Argentinian *B. rapa* accessions and try to infer the origin of herbicide-resistant populations. We analysed 56 Argentinian accessions and compared them with 568 accessions worldwide using genotyping-by-sequencing. A total of 15 790 SNPs were used to analyse genetic diversity, population structure, and phylogeny. Resistant and susceptible Argentinian accessions formed a genetically homogeneous group distinct from global populations. An exception was a population from southwestern Argentina, which clustered separately. The lowest genetic differentiation was observed with weedy strains from South and North America and with European turnips, suggesting a shared origin and local differentiation. The genetic similarity between resistant and susceptible accessions suggests that herbicide resistance may have emerged via local gene flow from transgenic *Brassica napus* cultivated informally in Argentina. Overall, our findings improve understanding of the genetic diversity and evolutionary history of this weed and provide a genomic baseline for future management strategies, as well as insights into transgene escape.

## Teaser

Feral plants can evolve from crops and become problematic weeds. In Argentina, wild populations of Brassica rapa have rapidly expanded and herbicide-resistant populations have emerged. By analyzing genomic data from local and global samples, we found that Argentinian populations are genetically similar and likely share a common origin. Herbicide resistance appears to have evolved locally, possibly through gene flow from cultivated relatives, highlighting ongoing crop–weed interactions.

## Introduction

Interspecific hybridization between crops and their wild relatives can occur through gene flow, enabling the transfer of domesticated traits into wild populations (Ellstrand *et al.* 2013). This process raises environmental concerns regarding the introgression of crop alleles—particularly transgenes—into wild species, potentially leading to ecological consequences such as the unintended spread of herbicide-resistant traits into natural ecosystems (Turnbull *et al.* 2021, Sohn *et al.* 2022). Feral plants, originating from escaped cultivated individuals, can establish self-sustaining populations and act as genetic intermediaries between crops and wild or weedy relatives, potentially facilitating the unintentional movement of crop-derived alleles across agroecosystems (Gressel 2005). These processes are especially relevant in closely related crop–weed complexes such as *Brassica rapa*, where gene exchange may enhance weediness traits, making this species a valuable model for studying domestication, ferality, and invasion.

*Brassica rapa* L. is a *Brassicaceae* species comprising wild, feral, and domesticated forms. Free-living forms have been grouped into subsp. *sylvestris* regardless of whether they are truly wild or feral crops (McAlvay *et al.* 2021). In some countries these forms are classified as noxious weeds (Gulden *et al.* 2008) and are commonly found in disturbed habitats, including roadsides, waste areas, farmlands, and riversides, throughout temperate regions (Wilkinson *et al.* 2003, Hall *et al.* 2005, Andersen *et al.* 2009). Domesticated forms have undergone extensive diversification, resulting in several subspecies grouped into three main categories: oleiferous types, leaf or flower vegetables, and turnip types. The turnip category consists of subsp. *rapa*, while the oleiferous group includes subspecies such as subsp. *trilocularis* (yellow sarson), subsp. *dichotoma* (brown sarson), and subsp. *oleifera* (oilseed field mustard), which are primarily grown for oil production. The leaf and flower types encompass subsp. *chinensis* (bok choy), subsp. *pekinensis* (napa cabbage), and European vegetables such as grelos (Spain) and rapini (Italy). Other members of this group include subsp. *parachinensis*, subsp. *narinosa*, subsp. *nipposinica*, and subsp. *perviridis* (Prakash *et al.* 2011, Bird *et al.* 2017).

The origin of *B. rapa* has been the subject of extensive debate. Previous studies suggested that the centre of origin of *B. rapa* was exclusively in Europe (Song *et al.* 1990, McGrath and Quiros 1992). However, a study conducted by Guo *et al.* (2014) proposed a broader theory, suggesting that the centre of origin of *B. rapa* also includes Western Asia and North Africa. *Brassica rapa* may have been the first diploid species of the *Brassica* genus to be domesticated, several millennia ago (Prakash *et al*. 2011). A recent study suggests that domesticated forms of *B. rapa*, including turnip and oilseed types, share a common origin in the mountains of Central Asia, dating back approximately 3430–5930 years. Following this initial domestication, selection for various forms, such as turnips, leafy vegetables, and oilseeds, took place, sometimes independently in parallel, across Eurasia (McAlvay *et al*. 2021).

*Brassica napus* (AACC, 2*n* = 4*x* = 38), commonly known as oilseed rape, is an allotetraploid crop that originated from interspecific crosses between the diploid species *B. rapa* (AA, 2*n* = 2*x* = 20) and *B. oleracea* (CC, 2*n* = 2*x* = 18). *Brassica napus* is one of the world’s leading oil crops, with a global production of 87 million tonnes (FAOSTAT 2025), as well as a root crop (rutabaga) and leaf crop (Siberian kale). Breeding programmes in Canada and the United States have developed oilseed varieties suitable for human consumption such as canola, including both transgenic and non-transgenic cultivars with herbicide resistance (HR). Approved transgenic herbicide-resistant cultivars include varieties resistant to glufosinate, glyphosate, and bromoxynil (Green 2009). Non-transgenic cultivars encompass resistance to AHAS-inhibiting herbicides, developed through mutagenesis and marketed as Clearfield^®^ oilseed rape, as well as those resistant to triazine herbicides, generated via natural mutation followed by backcrossing (Tan *et al.* 2005, Krato and Petersen 2012).

Transgenic feral *B. napus* populations have been reported in regions where the cultivation of transgenic rapeseed is authorized, such as Canada and the United States (Yoshimura *et al.* 2006, Schafer *et al.* 2011, Devos *et al.* 2012). In these regions, hybrids between *B. napus* and wild *B. rapa* have also been documented, facilitated by their sexual compatibility and some of these hybrids have inherited the glyphosate resistance trait (Warwick *et al.* 2003, 2008, Simard *et al.* 2006, Yoshimura *et al*. 2006, Aono *et al.* 2011). Experimental studies have confirmed that gene flow from transgenic *B. napus* to *B. rapa* is possible via hybridization, leading to the introgression of transgenes into *B. rapa* populations. These studies further indicate that at least three generations of backcrossing are required to stabilize the transgene in *B. rapa*, yielding fertile progeny that retains the herbicide-resistance trait (Pandian *et al.* 2024).

In Argentina, the cultivation of transgenic oilseed rape has been banned since 1997 due to concerns about gene flow to wild relatives, which are widely distributed across the country. Nevertheless, in 2012, populations of *B. rapa* exhibiting resistance to both glyphosate (conferred by a transgene) and AHAS-inhibiting herbicides were identified in glyphosate-resistant transgenic soybean fields (Pandolfo *et al.* 2016, 2018). Until then, *B. rapa* was not considered a major agronomic problem, as it could be effectively controlled by farmers. However, since the initial detection, these herbicide-resistant *B. rapa* biotypes have spread extensively, now affecting more than one million hectares across 116 counties in 7 provinces, representing a significant agronomic challenge in the country (AAPRESID 2023).

Previous research has shown that the acquisition of resistance genes to glyphosate and AHAS-inhibiting herbicides does not negatively impact the fitness of the Argentinian *B. rapa* accessions. This finding suggests that these traits may persist in the environment over time, particularly under strong selective pressure from herbicide use (Tillería *et al.* 2024). However, despite the increasing importance of *B. rapa* as a weed, little is known about the genetic diversity, structure, and evolutionary origin of the populations in the country, including herbicide-resistant biotypes. This lack of information limits our ability to understand how local populations have arisen and how they are related to global *B. rapa* diversity.

Genetic analyses involving crop–wild relatives can provide insights into the contributions of wild or feral populations to domesticated lineages through introgression (Cornille *et al.* 2012), or the dynamics of wild–weedy–domesticated species complexes (Zizumbo-Villarreal *et al.* 2005). While large diversity sets of global *B. rapa* populations have been studied using genotyping-by-sequencing (GBS) (Bird *et al.* 2017, McAlvay *et al.* 2021), these studies included only a single Argentinian sample collected in 1948, along with several others from different parts of South America. Their findings suggest that South American *B. rapa* populations are likely feral, rather than truly wild. However, to date, no studies have evaluated the genetic diversity of feral field mustard populations in Argentina, including those reported since 2012 that exhibit resistance to multiple herbicides and have become a major agricultural pest.

The aim of this study was to investigate the genetic diversity and population structure of *B. rapa* accessions collected across Argentina and to trace the origin of weedy populations by comparing them with accessions from diverse regions worldwide. Understanding the genetic background and evolutionary history of these populations is essential for developing strategies to limit their spread, while also providing a valuable model for studying transgene escape and its evolutionary consequences in other systems. To this end, we employed a GBS approach to generate genome-wide single nucleotide polymorphisms (SNPs), which allowed us to characterize the genetic diversity of Argentine accessions and compare them with previously published datasets of *B. rapa* representing multiple geographical regions and subspecies. This study addresses three key questions: (i) What is the genetic diversity and population structure of *B. rapa* in Argentina? (ii) Does SNP variation suggest a single introduction event or multiple independent introductions? and (iii) What is the most likely origin of the transgenic *B. rapa* biotypes found in the country?

## Materials and methods

### Sampling

**Figure 1:**
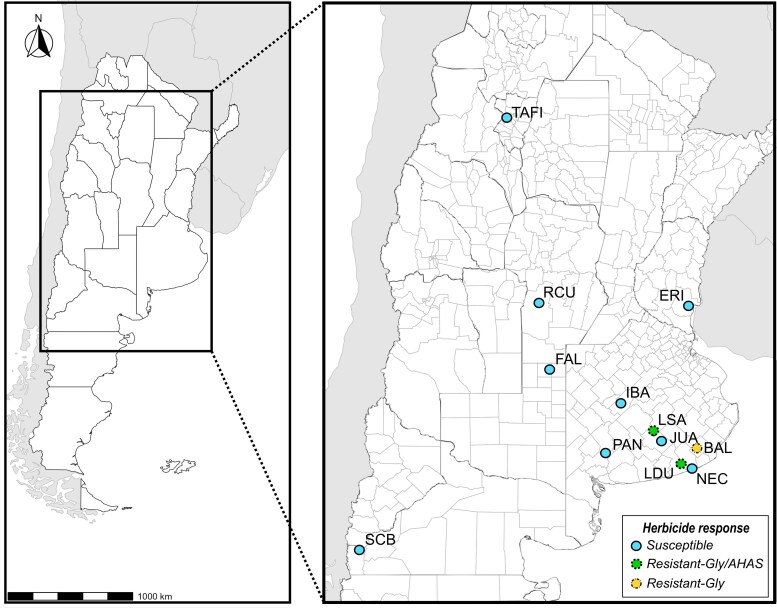
Geographic location of the *Brassica rapa* populations analysed in this study across Argentina. Dots represent sampled populations, with colours indicating their herbicide response: light blue for susceptible, green for glyphosate and AHAS-resistant, and yellow for glyphosate-resistant individuals (Pandolfo *et al.* 2016, Tillería *et al.* 2024).

We analysed 68 weedy *B. rapa* samples collected from different locations across six provinces of Argentina. The sampling sites included Tafí del Valle county in Tucumán province, Río Cuarto in Córdoba province, Falucho in La Pampa province, Concepción del Uruguay in Entre Ríos province, San Carlos de Bariloche (SCB) in Río Negro province, and seven locations in Buenos Aires province: La Sarita, La Dulce, Juarez, Balcarce, Necochea, Ibarra and Arroyo El Pantanoso (Sierra de la Ventana) (Table 1, Fig. 1).

**Table 1:** Sampling information and population genetic diversity estimates for Argentinian *Brassica rapa* populations.

Origin	ID	Year	Lat.	Long.	*n*	*π*	Tajima’s *D*	Features
La Dulce, Argentina	LDU	2012	−38.3569	−58.9892	3	0.00180	−1.2841	Within an agricultural field. Transgenic resistance to glyphosate and resistance to AHAS.
La Dulce, Argentina	LDU	2018	−38.3569	−58.9892	5	0.00255	0.1663	Transgenic resistance to glyphosate and resistance to AHAS.
Tafí del Valle, Argentina	TAFI	2009	−26.8839	−65.6917	5	0.00227	0.0452	—
Río Cuarto, Argentina	RCU	2009	−33.0053	−64.4039	5	0.00260	−0.0322	—
Balcarce, Argentina	BAL	2008	−37.5178	−58.8444	5	0.00256	0.0882	—
Balcarce, Argentina	BAL	2019	−37.5178	−58.8444	5	0.00274	0.0506	—
Balcarce, Argentina	BAL	2013	−37.5903	−58.5330	5	0.00195	−0.4236	Within an agricultural field. Transgenic resistance to glyphosate.
Necochea, Argentina	NEC	2009	−38.5455	−59.2403	5	0.00157	−1.6714	On the edge of road 228.
Benito Juarez, Argentina	JUA	2010	−37.6167	−59.6408	5	0.00247	0.2004	Uncultivated lowland on the edge of road 74.
Bariloche, Argentina	SCB	2017	−41.0525	−71.5311	5	0.00248	0.1814	On the edge of the lake Nahuel Huapi.
Concepción del Uruguay Argentina	ERI	2011	−33.1005	−58.6225	5	0.00263	0.0111	On the edge road 14.
Falucho, Argentina	FAL	2010	−35.1825	−64.0108	5	0.00260	−0.0199	—
Arroyo El Pantanoso, Argentina	PAN	2009	−37.9455	−61.8925	5	0.00271	−0.0295	—
Azul, Est. La Sarita, Argentina	LSA	2016	−37.1303	−60.0069	5	0.00244	−0.0566	Within an agricultural field. Transgenic resistance to glyphosate and resistance to AHAS.

The ID column corresponds to the internal code used to identify populations across all analyses and figures, and the *n* column corresponds to the number of individuals analysed for each population. Lat, Long: latitude and longitude of collection site. Nucleotide diversity (*π*) and Tajima’s *D* were estimated in 100-kb windows for each population. The resistance status was previously reported in our earlier studies (Pandolfo *et al.* 2016, Tillería *et al.* 2024).

We included accessions collected from the same locations across different years to assess change over time in populations (Table 1). In Buenos Aires province, the population from La Dulce town (LDU) included accessions collected in 2012 and 2018. Both accessions were collected within a soybean field and showed glyphosate and AHAS-inhibiting HR. For Balcarce county (BAL), samples were collected in three different years. The accessions from 2008 and 2019 were collected on the edge of a soybean field, whereas the accession from 2013, which exhibited glyphosate resistance, was within the field. The presence of the CP4 EPSPS enzyme from *Agrobacterium tumefaciens* was previously confirmed in the glyphosate-resistance accessions using herbicide trials, immunological tests, and specific molecular markers (Pandolfo *et al.* 2018, Tillería *et al.* 2024). AHAS-resistant biotypes were confirmed with herbicide assays and specific CAPs markers for the Trp574Leu mutation (Torres Carbonell *et al.* 2020).

### DNA extraction and sequence analysis

Seeds from each accession were sown in germination pots and grown in a greenhouse. Young leaves from seedlings were collected for total genomic DNA extraction, following the CTAB-based method (Doyle and Doyle 1987). The GBS libraries of the *B. rapa* samples were constructed using the ApeKI restriction enzyme, a common adapter, and standardized barcodes (Elshire *et al.* 2011). The samples were pooled in a 96-plex format and sequenced on an Illumina HiSeq 2000 platform (Illumina Inc., San Diego, CA) at the University of Wisconsin Biotechnology Center (UWBC). Raw reads of Argentinian samples will be submitted to the NCBI Short Read Archive (SRA) upon manuscript acceptance.

To infer the origin of Argentinian *B. rapa* accessions, we included individuals with previously published GBS data from Bird *et al.* (2017) and McAlvay *et al.* (2021). These samples comprise cultivated and weedy *B. rapa* biotypes from different parts of the world, including leafy forms, napa cabbage (*B. rapa* ssp. *pekinensis*), bok choy (*B. rapa* ssp. *chinensis*), mizuna (*B. rapa* ssp*. nipposinica*), tatsoi (*B. rapa* ssp. *narinosa*), choy sum (*B. rapa* ssp. *parachinensis*), rapini and grelos (*B. rapa* ssp. *sylvestris* var. *esculenta*) komatsuna (*B. rapa* ssp. *perviridis*), and weedy (*B. rapa* ssp. *sylvestris*); oleiferous types such as oilseed rape (*B. rapa* ssp. *oleifera*), yellow seeds/yellow sarson (*B. rapa* ssp. *trilocularis*), brown sarson/toria (*B. rapa* ssp. *dichotoma*) and the turnip forms (*B. rapa* ssp. *rapa*) (Supplementary Table S1). We also included a *Brassica oleracea* sample as an outgroup for our analysis. Raw reads were processed using STACKS pipeline (Rochette *et al.* 2019). The obtained reads were aligned to the *B. rapa* Chiifu v4.0 reference genome (Zhang *et al.* 2023) using the Burrows–Wheeler Aligner (BWA) v0.7.17 with the BWA–MEM algorithm (Li and Durbin 2009). The -M option was used to mark shorter split hits as secondary alignments, while all other parameters were kept at default settings. SAM files were converted to BAM format and sorted using SAMtools. SNP calling and genotyping were performed using the gstacks module in STACKS, and the final VCF file was generated using the populations module.

The final VCF file, which includes genotypes from Argentinian populations as well as cultivated and weedy biotypes from different parts of the world, was filtered using VCFtools, to retain only high-quality SNPs for the analysis (Danecek *et al.* 2011). For all analyses, we included only biallelic SNPs with a minimum mean depth of 5, a minimum genotype quality of 20, and minimum genotype call rate of 60% per site. A minor allele frequency threshold of 5% was applied for all analyses. The final VCF file is deposited in Figshare (https://doi.org/10.6084/m9.figshare.31906834).

### Definition of population groups

To investigate the genetic relationships and structure among *B. rapa* populations, we categorized accessions based on their growth form—wild (Caucasus, Italy, Siberia), weedy/feral, and cultivated types (oilseed field mustard, turnip, toria, brown sarson, bok choy, grelos, tatsoi, napa cabbage, rapini, komatsuna, choy sum, mizuna, yellow sarson). We also considered their geographical origin, primarily organized by continent, with a few exceptions. The Americas were subdivided into North and South America, and within these, two specific groups were treated separately: samples from Mexico were excluded from the North American group and considered a distinct unit due to their large sample size, and Argentinian samples were analysed independently of the broader South American group given their relevance to this study (Supplementary Table S2). Additionally, the Caucasus was treated as a separate region. Although not a continent, it represents a transcontinental area between Eastern Europe and Western Asia, often considered a biogeographic transition zone. This region is also where truly wild *B. rapa* types have been identified (McAlvay *et al.* 2021).

### Diversity and genetic structure

To determine genetic differentiation between populations, we calculated the fixation index (*F*_st_) using Arlequin v3.5 (Excoffier and Lischer 2010). This parameter was measured according to Weir and Cockerham (1984).

To investigate the genetic structure of *B. rapa* populations, we first conducted a Bayesian clustering analysis, including only Argentinian samples and testing *K* values from 1 to 15. We then extended the analysis to the global dataset, evaluating a range of genetic clusters (*K*) from 1 to 20. In both analyses, the optimal *K*, defined as the value that maximized the marginal likelihood, was identified using the ChooseK script included in the fastStructure v1.0 package (Raj *et al.* 2014). Bar plots for different *K* values were visualized using the POPHELPER R package (Francis 2017).

Additionally, we conducted a principal component analysis (PCA) using the R package SNPRelate (Zheng *et al.* 2012) to evaluate the genetic structure of the populations, and the results were visualized using the ggplot2 package (Wickham 2017).

To assess genetic diversity within populations, we determined heterozygosity rates and the proportion of missing data using the genotype module in TASSEL 5 (Glaubitz *et al.* 2014). Nucleotide diversity (*π*) was calculated in non-overlapping 100-kb windows, using pixy v2.0.0.beta14 (Korunes and Samuk 2021), with an all-sites VCF including both variant and invariant sites as input. Tajima’s *D* was also calculated in non-overlapping 100-kb windows using pixy v2.0.0.beta14 for the Argentinian accessions (Bailey *et al.* 2025).

### Phylogenetic inference

To infer the origin of the herbicide-resistant *B. rapa* accessions from Argentina and to visualize their hierarchical relationships, we constructed a neighbor-joining (NJ) tree based on pairwise genetic distances estimated from SNP data using VCF2Dis (Xu 2025). Additionally, we built a maximum likelihood (ML) tree using RAxML 8.2.12 (Stamatakis 2014), employing rapid bootstrapping with 100 replicates under the ASC_GTRGAMMA model with Lewis correction for ascertainment bias (Lewis 2001), appropriate for SNP datasets lacking invariant sites. Both resulting trees were plotted with the ggtree (Yu *et al.* 2017) R package.

To further explore species relationships, we applied a coalescent-based approach using SVDquartets, implemented in PAUP 4.0 (Swofford 2003) on the CIPRES platform (Miller *et al.* 2012). This analysis inferred relationships among quartets of taxa under the Multispecies Coalescent (MSC) model, based on site pattern analysis. We evaluated all possible quartets and generated 100 bootstrap replicates, assembling the resulting quartet trees to estimate a species tree grouped by crop type and geographical region. The SVDquartets tree was visualized using iTOL (Letunic and Bork 2024). The three trees were rooted with a *B. oleracea* sample.

## Results

### SNP calling and SNP filtering

After filtering the VCF file, we retained 56 *B. rapa* accessions from Argentina of the 68 originally analysed, as samples with more than 50% missing genotype data were excluded, and identified a total of 27 612 SNPs through GBS. Upon analysing previously published GBS data, we retain a total of 15 790 SNPs from 624 samples, including the 56 samples from Argentina, with the remaining representing accessions from various regions worldwide.

### Genetic diversity and structure

Nucleotide diversity (*π*) between Argentinian accessions varied moderately among accessions, ranging from 0.00157 (NEC) to 0.00274 (BAL19) (Table 1). Most accessions showed Tajima’s *D* values close to zero, consistent with neutral expectations. However, some accessions exhibited markedly negative Tajima’s *D* values, particularly LDU12 (*D* = −1.28) and NEC (*D* = −1.67), whereas others showed slightly positive values, such as JUA (*D* = 0.20) and SCB (*D* = 0.18).

**Figure 2:**
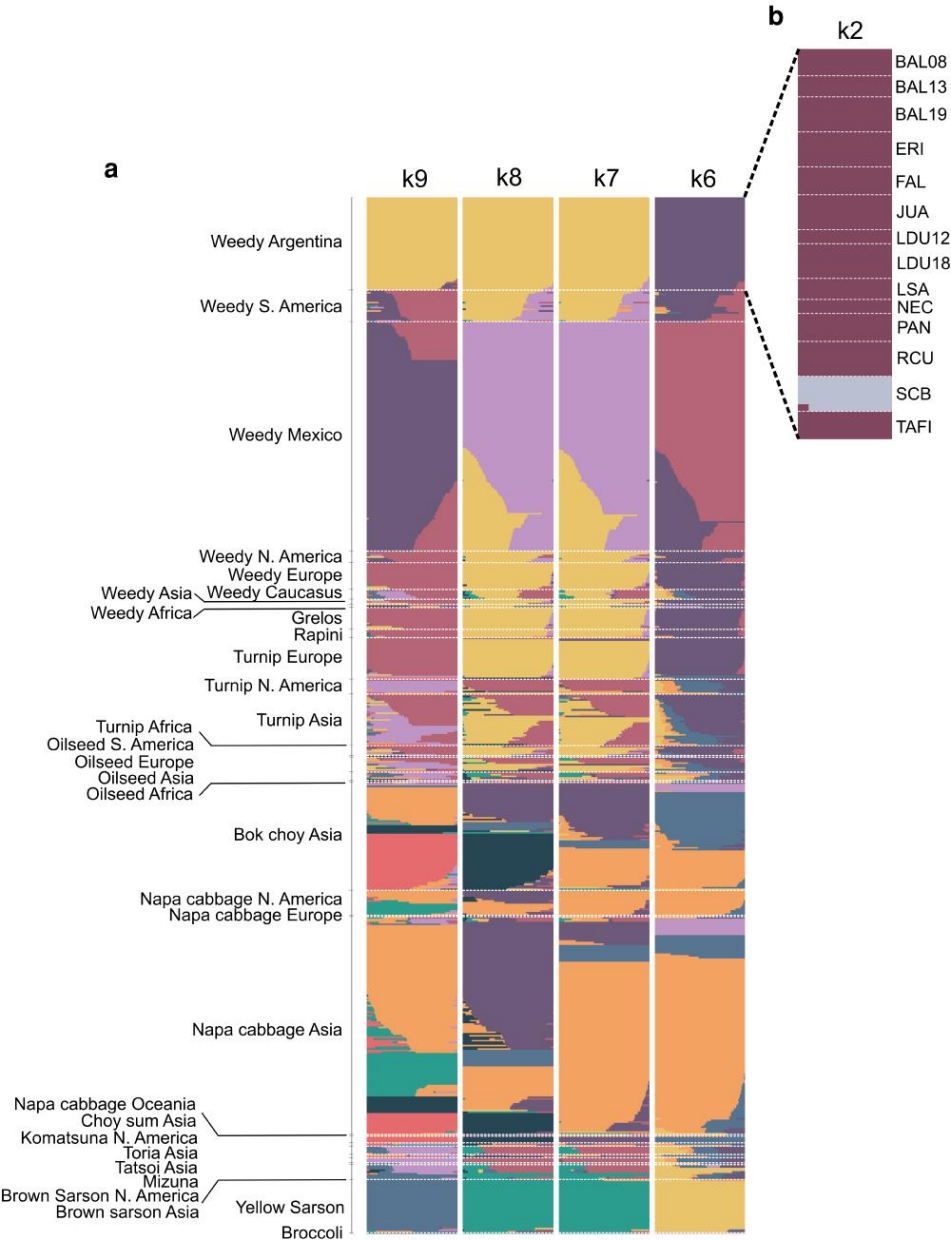
a) FastStructure plot showing the population structure of crops and weedy accessions of *Brassica rapa* from different regions worldwide, at four different values of *K* (*K* = 6 to *K* = 9). Sample labels correspond to accessions grouped by biotype and geographic origin. b) FastStructure plot showing the population structure of Argentinian *Brassica rapa* accessions at *K* = 2.

FastStructure analysis of Argentinian accessions, performed across *K* values ranging from 1 to 15, the ChooseK script identified *K* = 2 as the value that maximized the variational marginal likelihood (Supplementary Fig. S1, Supplementary Table S3). At *K* = 2, all Argentine samples shared a common ancestry component, while the SCB accession was clearly differentiated from the remaining accessions, with no evidence of admixture (Fig. 2b). At *K* = 3, BAL08 and JUA formed a distinct genetic cluster, whereas SCB and TAFI grouped into a separate ancestry component, and the remaining accessions constituted a third cluster. At this level of clustering, some individuals from the BAL08 and TAFI accessions showed evidence of admixture in their genetic structure (Supplementary Fig. S2).

Using the global dataset, the analysis of nucleotide diversity (*π*) within groups, categorized by crop type and geographical region, revealed that the weedy Argentinian biotype exhibited low nucleotide diversity (*π* = 0.0011492031) compared with weedy and cultivated types from other regions. In contrast, the highest diversity was found in the cultivated group of oilseed rape and brown sarson from Asia (*π* > 0.004). The weedy forms showed intermediate values, ranging from 0.0020 to 0.0029 (Table 2).

**Table 2:** Number of samples (*n*) and nucleotide diversity (*π*) of *Brassica rapa* groups categorized by crop type and geographical region.

Morphotype	Group	*n*	*π*
Leafy	Bok choy Asia	65	0.0034557664
Leafy	Napa cabbage Asia	131	0.0031405939
Leafy	Grelos	13	0.0022452331
Leafy	Rapini	5	0.0012777609
Leafy	Napa cabbage North America	15	0.0011838523
Leafy	Mizuna Asia	3	0.0009182736
Leafy	Tatsoi Asia	2	0.0007959671
Leafy	Choy sum Asia	4	0.0003502452
Oilseed	Oilseed rape Asia	5	0.0040973011
Oilseed	Brown sarson Asia	9	0.0040538599
Oilseed	Toria Asia	5	0.0020673361
Oilseed	Oilseed rape Europe	9	0.0018881779
Oilseed	Yellow Sarson	32	0.0004343933
Turnip	Turnip North America	9	0.0024322024
Turnip	Turnip Europe	25	0.0023377494
Turnip	Turnip Asia	31	0.0023170715
Turnip	Turnip Africa	6	0.0013534852
Weedy	Weedy South America	19	0.0029539637
Weedy	Weedy North America	7	0.0026985749
Weedy	Weedy Africa	2	0.0025488530
Weedy	Weedy Europe	16	0.0025269318
Weedy	Weedy Mexico	138	0.0020073325
Weedy	Weedy Argentina	56	0.0011492031
Weedy	Weedy Asia	3	0.0007757952
Wild	Wild Caucasus	6	0.0017973856

After performing the FastStructure analysis across *K* values ranging from 1 to 20, the ChooseK script identified a *K* value of nine as the value that maximized the marginal likelihood (Supplementary Fig. S3, Supplementary Table S4). The plot from *K* = 6 to *K* = 9 (Fig. 2a) shows that the Argentinian accessions, when compared with accessions from the rest of the world, form a homogeneous cluster. Admixture was observed only in the SCB accessions at *K* = 6 to *K* = 9, with ancestral components shared with the weedy biotype of South America and North America. At *K* = 6, Argentinian samples shared a significant proportion of ancestry with feral biotypes from the Americas, Europe, Asia, and Africa, as well as with the European biotypes of grelos, rapini, and turnip. At *K* = 7 and *K* = 8, the Argentinian samples continued to share substantial ancestry with these biotypes but showed differentiation from weedy samples from México. At *K* = 9, the Argentinian samples formed a distinct group, clearly separated from the rest of the accessions. The oilseed biotypes show high levels of admixture across all evaluated *K* values. In the case of bok choy and napa cabbage accessions, they show a different ancestry compared to the rest of the *B. rapa* samples, clustering only with each other. Meanwhile, the biotype of *B. rapa* yellow sarson consistently formed a distinct group across all evaluated *K* values, without sharing any ancestry with other accessions worldwide.

### Phylogenetic analyses

**Figure 3:**
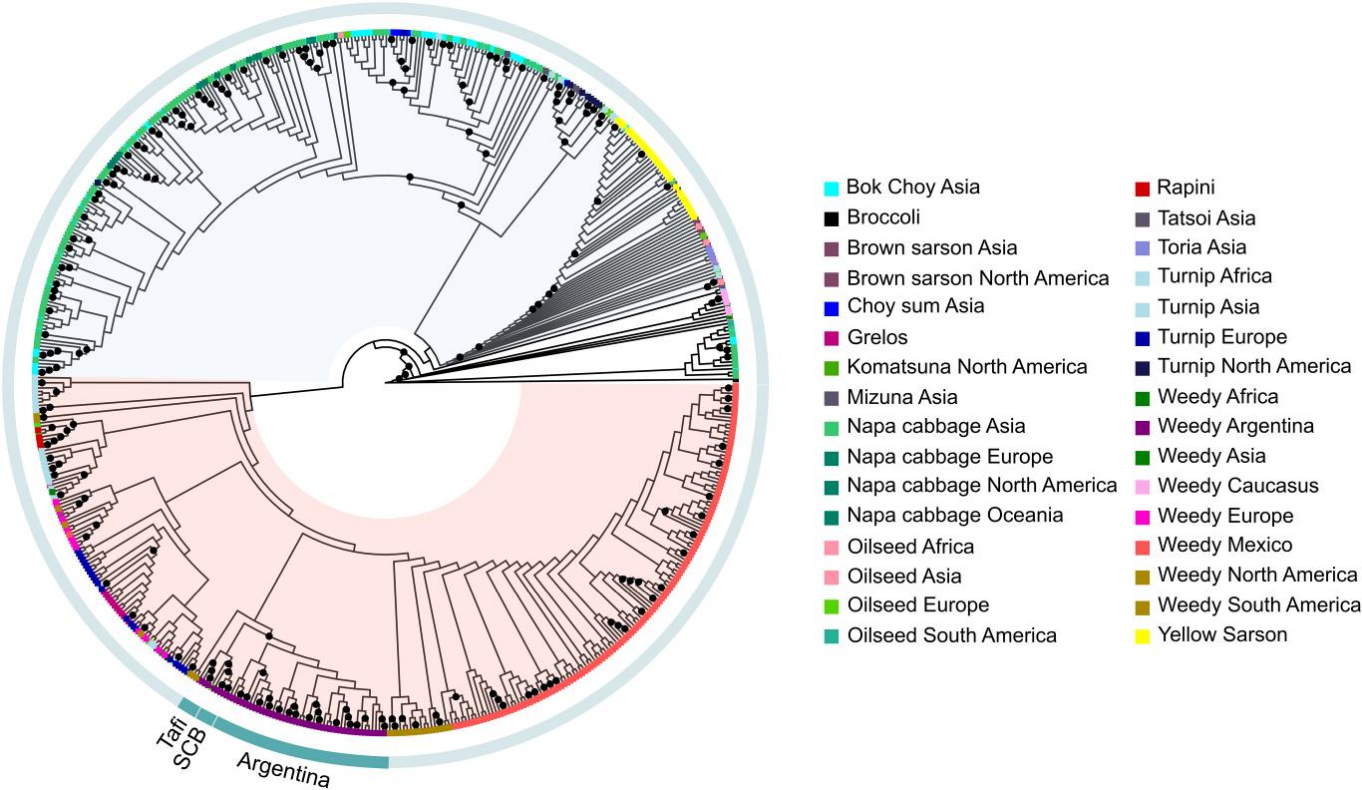
Maximum likelihood tree (RAxML) of 624 *Brassica rapa* individuals, including 56 Argentinian samples and 568 accessions from around the world. Colours represent different morphotypes and geographic origins, including weedy populations and cultivated forms. Black circles on nodes represent bootstrap support; only values equal or greater than 90% are shown. The pink background highlights a clade that includes weedy forms and some cultivated types (turnip Europe, rapini, grelos), while the light blue background marks a clade containing the remaining cultivated forms. The label ‘Argentina’ identifies the clade that contains all Argentinian samples except those from San Carlos de Bariloche (SCB) and Tafí del Valle (TAFI), which cluster in separate clades labelled ‘SCB’ and ‘TAFI’, respectively.

Phylogenetic relationships among samples were inferred using both a maximum-likelihood approach (RAxML) and a distance-based NJ method. The maximum-likelihood tree was structured into two main clades, a pattern also observed in the NJ tree (Fig. 3, Supplementary Fig. S5). One clade included individuals belonging to cultivated forms, containing the bok choy, napa cabbage, tatsoi, mizuna, choy sum, komatsuna, yellow sarson, and brown sarson accessions. The second major clade comprised weedy forms, along with the cultivated forms of turnip, grelos, and rapini from *B. rapa*. Within this second clade, the RAxML tree revealed that the Argentinian samples clustered together in a distinct clade, except for the SCB and TAFI population, which grouped separately as a sister clade. The Argentinian clade, together with the wild forms from these regions, formed a well-supported group that was sister to another clade comprising wild forms from Asia and the Caucasus, as well as cultivated forms of turnip, grelos, and rapini.

Similarly to the RAxML tree, the NJ tree showed that all Argentinian accessions clustered together, except for the SCB accessions. These individuals clustered separately with a sample of the oilseed biotype from Europe, next to the weedy/feral forms from North America and South America (Supplementary Fig. S4). We observed that, in a major clade, the Argentinian samples were grouped with the weedy accessions from South America, which included samples from Chile, Peru, Bolivia, and Colombia, along with most of the Mexican weedy samples.

**Figure 4:**
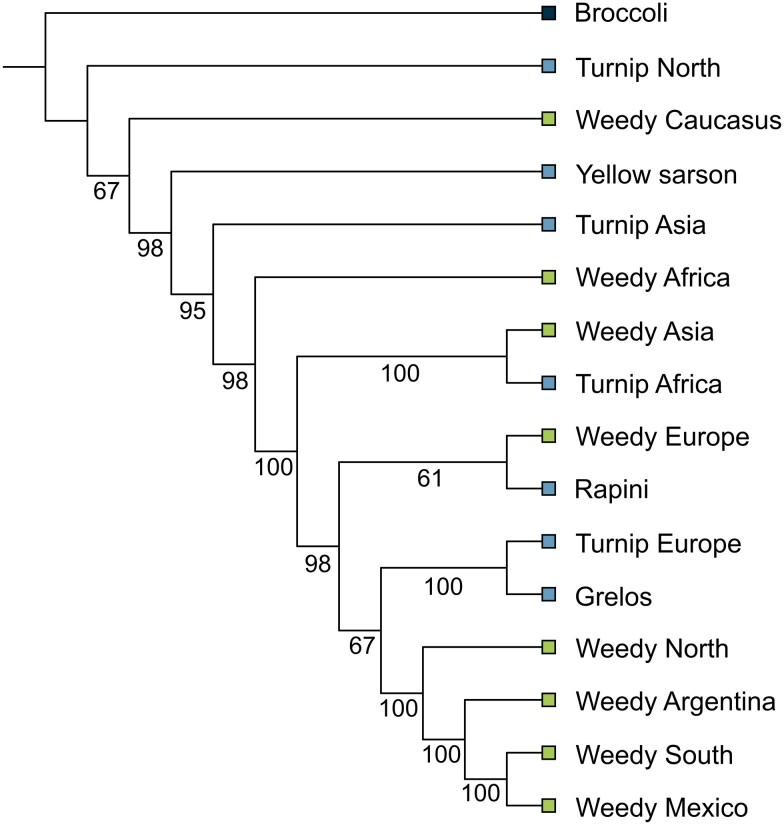
SVDquartets coalescent species tree of *Brassica rapa* accessions grouped by crop type and geographic region. Bootstrap support values ≥61% are shown next to nodes. Label colours indicate biotype: cultivated forms (blue) and weedy forms (green).

The SVDquartets coalescent tree revealed that the Argentinian biotypes clustered with weedy biotypes from the Americas (Fig. 4). At a higher hierarchical level, these biotypes—including the Argentinian ones—were grouped with turnip and weedy biotypes from Europe. Low bootstrap support was found at the base of this clade, suggesting that the differences between these groups are not strong enough to support their separation with high confidence. These results are consistent with those obtained from the RAxML and NJ analyses, which also showed that Argentinian samples cluster with weedy biotypes from the Americas and are closely related to turnip and European weedy forms.

### Principal component analysis

**Figure 5:**
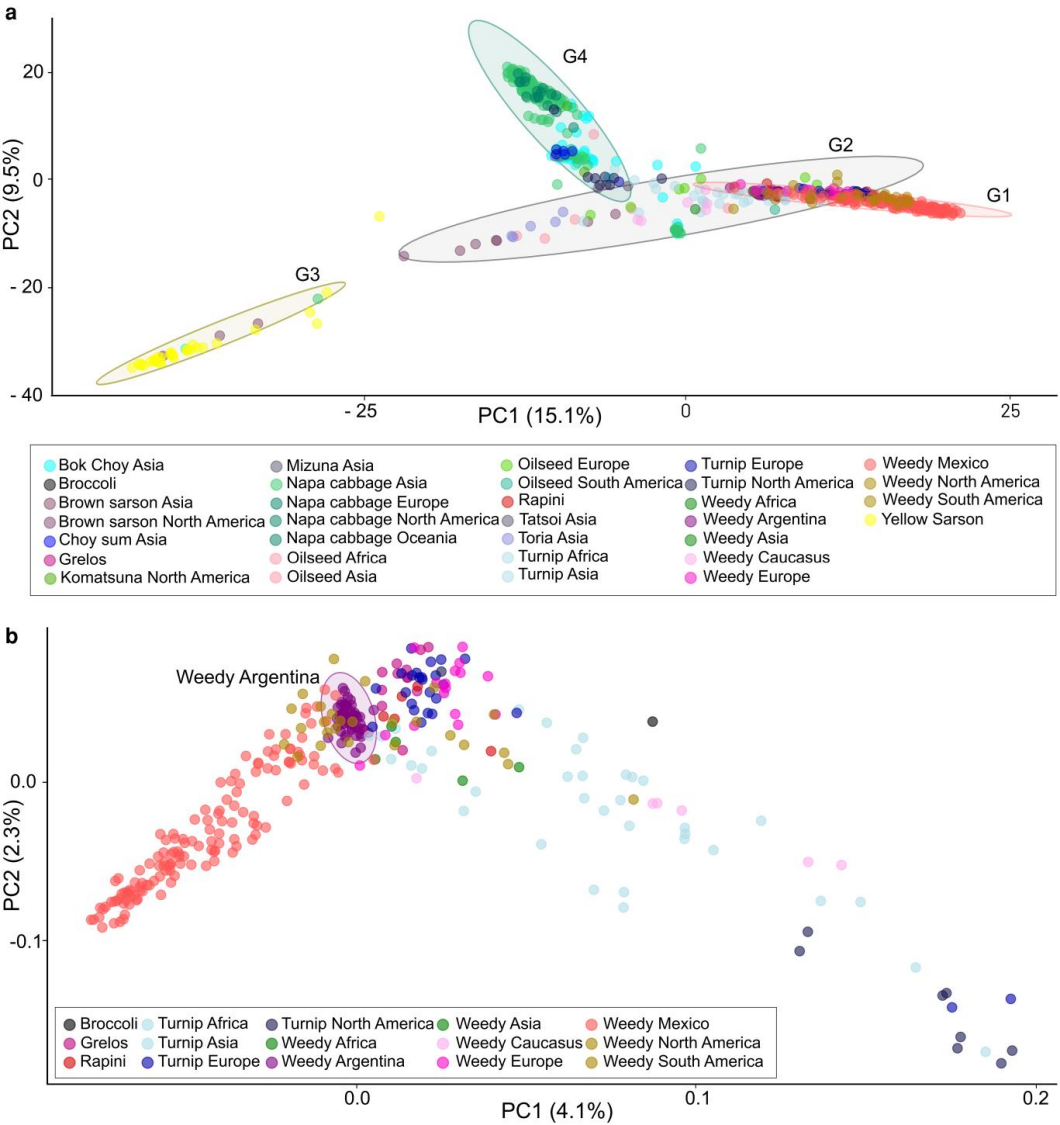
Principal component analysis (PCA) based on SNPs. Colours represent different morphotypes and geographic origins, including weedy populations and cultivated forms. a) Scatter plot of PC1 vs. PC2 showing genetic variation among 624 *Brassica rapa* accessions worldwide. Samples are grouped into four genetic clusters: G1 (weedy Argentina, grelos, oilseed Europe, oilseed South America, rapini, turnip Europe, weedy Africa, weedy Asia, weedy Europe, weedy Mexico, and weedy North America biotypes); G2 (brown sarson Asia, brown sarson North America, toria Asia, oilseed Asia, oilseed Europe, weedy Caucasus, oilseed Africa, and turnip Asia); G3 (yellow sarson biotype); G4 (bok choy Asia, tatsoi Asia, mizuna Asia, oilseed Europe, napa cabbage Asia, napa cabbage North America, napa cabbage Europe, napa cabbage Oceania, choy sum Asia, komatsuna North America, turnip Africa, and turnip North America). b) PCA was conducted on a restricted dataset comprising Argentinian weedy samples together with weedy biotypes from other regions, turnip, grelos, and rapini accessions; *B. oleracea* (broccoli) was included as an outgroup.

The PCA analysis of the 624 *B. rapa* samples revealed distinct clusters (Fig. 5a). We identified four main groups, in which the samples were grouped based on region and biotype. The Argentinian samples clustered together with weedy individuals (subsp. *sylvestris*) from Europe, South America, and North America (including those from Mexico), as well as with the grelos, rapini, and turnip biotypes from Europe. Samples from bok choy, napa cabbage, tatsoi, mizuna, choy sum, and komatsuna accessions were closely associated with each other. In contrast, yellow sarson individuals formed a distinct cluster, representing the most divergent group from the rest of the samples. To obtain a more detailed pattern, we restricted the analysis to include only the Argentinian samples and the closest groups. These results showed the Argentinian group closely associated with the weedy forms as well as with turnip from Europe, grelos, and rapini (Fig. 5b).

### Genetic differentiation

**Figure 6:**
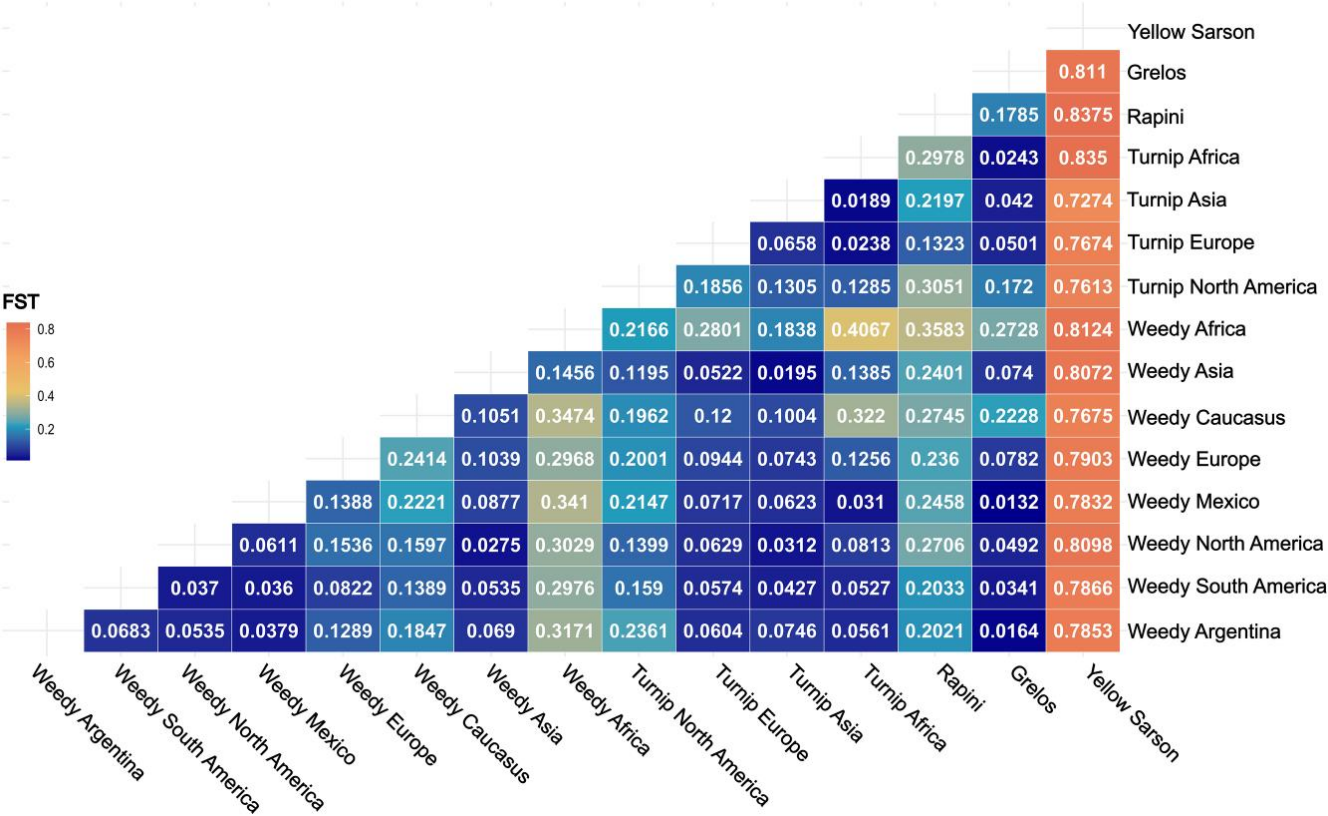
Heatmap of *F*_st_ values across *Brassica rapa* accessions categorized by crop type and geographical region.

We assessed the genetic differentiation (*F*_st_) between Argentinian *B. rapa* samples and weedy biotypes, as well as the turnip, rapini, and grelos biotypes. The yellow sarson group was included in the analysis as an outgroup. The lowest *F*_st_ value was observed between Argentinian samples and grelos biotype (*F*_st_ = 0.0164), followed by weedy forms from Mexico (*F*_st_ = 0.0379), North America (*F*_st_ = 0.0535), and South America (*F*_st_ = 0.0683). The highest genetic differentiation was observed between Argentinian samples and turnip from North America, wild forms from the Caucasus, and the rapini biotype (*F*_st_ > 0.18). As expected, the yellow sarson group showed a high level of genetic differentiation from all biotypes included in the *F*_st_ analysis (*F*_st_ > 0.7) (Fig. 6).

## Discussion

The recent expansion of *B. rapa* in Argentine agricultural fields, including populations resistant to both transgenic and non-transgenic herbicides, highlights the need to understand their genetic diversity and origins. Using a genome-wide dataset of 15 790 SNPs from 624 globally distributed accessions—including 56 newly sequenced Argentinian samples—we provide a global context for interpreting population structure and evolutionary relationships. Our results indicate that Argentinian accessions, irrespective of resistance status, form a genetically distinct group relative to global populations, suggesting a shared origin. An important exception is the susceptible population from SCB, which shows clear genetic differentiation, pointing to a distinct evolutionary history within Argentina.

### Genetic diversity and historical records support the feral nature of Argentinian *Brassica rapa* populations

Spontaneous forms of *B. rapa* (*Brassica rapa* subsp. *sylvestris*) have been considered by some authors to be the wild form of the species, for example, based on molecular characterization studies using SSR markers (Guo *et al.* 2014). However, other authors, through demographic analysis, found no evidence of differentiation from other subspecies, suggesting that it is not the wild form (Qi *et al.* 2017). Nucleotide diversity in the Argentinian group (*π* = 0.001149) was low compared to weedy and cultivated accessions from other regions, supporting the presence of a conserved gene pool. This pattern is congruent with recent findings suggesting that South American weedy forms of *B. rapa* are feral populations derived from cultivated ancestors, rather than true wild types, as evidenced by their low nucleotide diversity and close genetic relationship to turnips and European leafy crops (McAlvay *et al.* 2021). Earlier studies using RFLP markers also reached similar conclusions, identifying Argentinian weedy accessions as likely escapes from cultivation (Crouch *et al.* 1995).

De-domestication, the process by which crops become feral, generally leads to reduced diversity, often as a consequence of genetic bottlenecks associated with escape from domesticated crops and subsequent establishment in natural habitats (Gressel 2005). It is generally assumed that feral populations have lower nucleotide diversity than their wild relatives but higher than cultivated forms (Qiu *et al.* 2020, Hernández *et al.* 2022). In several species, genomic analyses have been used to distinguish truly wild populations from feral ones. For instance, in *B. oleracea*, multiple studies have shown that many presumed wild populations are in fact feral, as evidenced by their low levels of genetic diversity, close genetic distance to cultivated forms, and clustering with crop types in phylogenetic analyses (Maggioni *et al.* 2020, Mittell *et al.* 2020, Mabry *et al.* 2021). Another example, a study in feral populations of *B. napus* in Japan, has documented high genetic diversity in comparison with cultivated forms, revealing the dynamics of feralization (Chen *et al.* 2020). In our dataset, weedy accessions from Argentina, and from other regions, showed even lower diversity than cultivated forms such as Asian turnip (*π* = 0.002317), grelos (*π* = 0.002245), European turnip (*π* = 0.002337) and others. This suggests the occurrence of a strong bottleneck during the feralization process or post-domestication dispersal.

Historical records corroborate these genetic patterns. *Brassica rapa* has been reported in South America since the 16th century, likely introduced by Spanish explorers. During that period, *Brassica* species such as *B. napus* were commercialized for oil production, and *B. rapa* may have been introduced accidentally as a contaminant in seed lots (Bye 1979). At that time, turnip plants were harvested directly from wheat fields and irrigation ditches, where they grow spontaneously (Patiño 1963). In Argentina, *B. rapa* had become a prominent weed in cereal crops by the 1930s. Its naturalized form was so abundant in wheat and flax fields that the value of the harvested *B. rapa* seeds helped offset the cost of removing it from the grain and producing the cereal crop. As a result, the government promoted its cultivation, and for several decades, its seeds were traded in national markets and used in industrial oil production until the 1960s (Tenembaum 1937). Afterwards, the use of *B. rapa* seeds declined, and it was replaced by *B. napus* (Pascale 1976).

Today, only weedy forms of *B. rapa* persist in Argentina, while cultivated *Brassica* crops correspond almost exclusively to *B. napus* (Pascale 1976, FAOSTAT 2025). This history of local cultivation followed by abandonment may have shaped the unique genetic structure observed today in current Argentinian feral populations, in concordance with an endoferal origin, that is, populations derived from cultivated ancestors that subsequently escaped into local environments (Gressel 2005, Gering *et al.* 2019).

### Differentiated genetic structure of Argentinian *Brassica rapa* populations compared to worldwide populations

To place Argentinian samples in a global context, we conducted phylogenetic analyses using multiple methods, including maximum likelihood (RAxML), NJ, and SVDquartets. The RAxML analysis revealed two major clades. The first clade included the Argentinian accessions clustered with weedy/feral forms of *B. rapa* from various regions of the world, along with all samples of cultivated types such as European turnip, grelos, and rapini, which grouped exclusively with the weedy/feral forms—except for two European turnip samples that clustered within the cultivated clade. The second clade comprised the remaining cultivated accessions (Fig. 3). Although the diverse panel of Argentinian populations was not included in the study by McAlvay *et al.* (2021), their results likewise showed that American weedy forms clustered with European weedy and turnip types. Bird *et al.* (2017) also reported that the turnip biotype separates Asian and European forms, which is consistent with our findings.

Population structure analyses further supported these phylogenetic results. The fastStructure analysis showed that, at lower *K* values, the Argentinian accessions shared substantial ancestry with other weedy forms of different regions and cultivated biotypes principally with grelos, rapini and turnips forms from Europe (Fig. 2a). However, at *K* = 9, they appeared as a clearly differentiated group, forming a homogeneous cluster with no signs of admixture. In contrast, oilseed rape, bok choy, napa cabbage and choy sum accessions displayed a high degree of admixture across their samples, in agreement with recent whole-genome sequencing studies that report extensive gene flow in these groups (Saban *et al.* 2023).

Across phylogenetic and population structure analyses, the Argentinian population SCB consistently emerged as a genetic outlier. In the maximum-likelihood phylogeny, SCB and TAFI formed a sister clade to the remaining Argentinian accessions, clustering near weedy populations from South and North America, whereas NJ analyses supported SCB as the only clearly differentiated Argentinian population, with TAFI grouping with the remaining samples. Consistently, fastStructure analyses revealed strong admixture signals in SCB. At *K* = 9, most Argentinian accessions formed a homogeneous cluster, while SCB shared ancestry with other South American weedy populations. When restricted to Argentina, the optimal *K* was 2, again separating SCB from all other accessions (Fig. 2b). Together, these results indicate a distinct evolutionary history for SCB relative to other Argentinian *B. rapa* populations.

The genetic distinctiveness of SCB may reflect either an independent introduction event or a more recent colonization from neighbouring countries, such as Chile. Multiple feralization events have been documented in other weedy systems, including weedy rice populations in California (De Leon *et al*. 2019). Notably, SCB was officially founded only in 1902 (Biedma 1987), making it a relatively recent settlement compared to cities in Buenos Aires Province, which supports the hypothesis of a recent introduction rather than long-term geographic isolation. Overall, the concordant evidence from phylogenetic and structure analyses underscores the unique genetic profile of SCB within the broader *B. rapa* landscape in Argentina (Figs 2 and 3).

### The origin of weedy *Brassica rapa* populations of Argentina

The consistent clustering of Argentinian *B. rapa* samples with weedy biotypes from the Americas and certain European cultivated forms (turnip, grelos, and rapini) across phylogenetic, multivariate, and population structure analyses suggests a shared genetic background and possibly a common origin. Both coalescent and distance-based phylogenies, along with PCA, placed the Argentinian accessions within a broader group that includes American weedy forms and European cultivars. This pattern supports a potential historical connection between Argentinian populations and these lineages, likely resulting from gene flow or past introduction events. The low bootstrap support at deeper nodes in the coalescent trees may reflect recent divergence or ongoing admixture among these lineages. Moreover, the *F*_st_ values indicate low genetic differentiation between Argentinian accessions and weedy forms from the Americas and turnip from Europe, as well as the grelos and rapini, reinforcing their close genetic affinity (Fig. 6). These findings are in strong concordance with the results of McAlvay *et al.* (2021), who also reported a close genetic relationship between American weedy forms and the European turnip biotype.

Based on these results, we propose that Argentinian weedy *B. rapa* populations may have at least two distinct genetic origins. The SCB population appears genetically divergent from the remaining Argentinian accessions. This pattern is consistent with a history of geographic isolation or an independent introduction event. One plausible hypothesis is that SCB may have originated from a neighbouring region, such as Chile, given the geographic proximity and its genetic clustering with that biotype; however, additional sampling would be required to test this hypothesis explicitly. In contrast, the remaining Argentinian populations show lower levels of genetic differentiation from one another, suggesting a shared evolutionary history shaped primarily by local evolutionary processes and restricted gene flow with *B. rapa* populations from other regions. The TAFI accessions represent an intermediate case, displaying relatively low genetic differentiation in the phylogenetic analyses, which may reflect its geographic position and potential local adaptation. Previous studies have shown that multiple independent feralization events can occur and that feral populations may continue to evolve through interactions with local cultivars. Together with local adaptation and limited gene flow, these processes can lead to the emergence of distinct genetic pools within feral lineages (Mabry *et al.* 2023).

Although Argentinian *B. rapa* populations likely share a feral origin with other South American accessions, our results suggest that they have diverged over time, forming a unique genetic lineage. Supporting this idea, a historical Argentinian accession from 1948 clustered within the broader South American clade (Fig. 3), indicating genetic similarity. During that period, *B. rapa* was cultivated in Argentina for oil production (Tenembaum 1937). However, this historical accession grouped into a different subclade than current Argentine samples, suggesting that local populations have since followed a distinct evolutionary trajectory, diverging from other South American groups.

### Origin and spread of herbicide-resistant *Brassica rapa* biotypes in Argentina

To investigate the origin of the Argentinian resistant accessions, we propose two main hypotheses. Given the prohibition of transgenic oilseed rape cultivation in Argentina, the first hypothesis posits that resistant *B. napus* and/or *B. rapa* biotypes were introduced directly into the country through contamination of seed shipments, a process facilitated by the small seed size of both species, as reported in Japan and Europe (Aono *et al.* 2011, Squire *et al.* 2011). Under this scenario, hybridization between resistant *B. napus* and feral *B. rapa* may have occurred prior to their introduction in countries where transgenic *B. napus* cultivation is allowed and where weed–crop hybrids with HR have already been reported, such as in Canada (Simard *et al.* 2006, Warwick *et al.* 2008, Laforest *et al.* 2022, FAOSTAT 2025). These resistant biotypes may have subsequently introgressed into local feral *B. rapa* populations, giving rise to individuals with an Argentinian genetic background but carrying resistance traits acquired from external sources.

An alternative hypothesis we proposed is that herbicide-resistant *B. napus* commercial varieties, both transgenic and non-transgenic, may have been introduced into Argentina as contaminants in imported seed lots or that an illegal cultivation of resistant *B. napus* crop was carried out in the country. In this way the hybridization with feral *B. rapa* populations has occurred in Argentina leading to resistant biotypes. Hybridization between these *B. napus* individuals and local feral *B. rapa* populations has been demonstrated in Argentina and could have facilitated the introgression of resistance alleles, ultimately leading to the establishment of resistant feral *B. rapa* biotypes (Ureta *et al.* 2017, Torres Carbonell *et al.* 2020).

Genetic analyses of Argentinian *B. rapa* populations revealed no clear genetic differentiation between herbicide-resistant and susceptible accessions, supporting a shared local origin of resistance. Populations sampled from the same region before and after the emergence of resistance showed no detectable genetic differentiation (Table 1). Population genetic analyses restricted to Argentinian accessions further revealed low and highly similar nucleotide diversity across populations (*π* = 0.00157–0.00274), indicative of recent divergence, and Tajima’s *D* values, were close to zero for most populations, suggesting approximate neutrality and no strong genome-wide signals of recent bottlenecks or directional selection (Table 1). Two accessions (LDU12 and NEC) showed moderately to strongly negative Tajima’s *D* values and reduced nucleotide diversity compared to other Argentinian populations. This pattern is consistent with recent demographic or selective processes, potentially reflecting localized selection and/or recent introduction events. Notably, LDU12 corresponds to the accession in which the transgene was first detected; suggesting that these populations may represent an early stage in the emergence of herbicide-resistant biotypes in Argentina. Nucleotide diversity in a later-sampled population from the same locality (LDU18) was comparable to that observed in other Argentinian accessions, indicating a rapid recovery of genetic diversity following the establishment of HR.

In addition, Argentinian *B. rapa* shows no evidence of repeated introgressions from exotic lineages or widespread regional migration. The genetic structure of Argentine *B. rapa* populations does not closely resemble any biotype from other regions of the world, including countries where transgenic *B. napus* is widely grown or transgenic *B. rapa* and *B. napus* is found (Saji *et al.* 2005, Hecht *et al.* 2014, Pandolfo *et al.* 2018). Nonetheless, analyses revealed the closest similarities with weedy biotypes from Chile and North America, both of which cultivate transgenic *B. napus*. This raises the possibility that herbicide-resistant biotypes may have entered Argentina via seed contamination from these areas, subsequently hybridizing with local *B. rapa*. Introgression may have preserved HR genes due to herbicide pressure in agricultural fields, while the rest of the genome became admixed with local lineages through hybridization, gradually acquiring the genetic background of local populations in a short period of time. This process may have been facilitated by the phenotypic plasticity of invasive species, which allows it to adapt quickly to new environmental conditions (Vigueira *et al.* 2013, Vercellino *et al.* 2023).

Although seed-mediated introgression has often been proposed as the most likely explanation for the presence of resistant biotypes (Pandolfo *et al.* 2016, 2018), the molecular evidence presented here supports the alternative hypothesis. If resistant individuals had been introduced via contaminated seed from abroad, they would be expected to show admixture with foreign populations; however, resistant accessions remain genetically indistinguishable from the Argentinian gene pool, even in samples collected prior to the first detection of resistance. This pattern is more consistent with a local origin of resistance, most likely through gene flow from transgenic *B. napus* cultivated informally in Argentina, leading to the introgression of resistance alleles into feral *B. rapa* populations. The subsequent spread of resistance alleles across Argentina may have been further promoted by agricultural practices, particularly the extensive use of hired harvesting machinery, which has been shown to facilitate weed seed dispersal (Tourn *et al.* 2018). The transgenic *B. napus* individuals reported by Pandolfo *et al.* (2016) may represent escapes from such unregulated cultivation. Nevertheless, further studies based on whole-genome sequencing are required to more clearly resolve the origin of the transgenic-resistant populations found in Argentina. Such analyses would enable precise identification of the genomic regions harbouring transgenes and their comparison with commercial cultivars, thereby providing a more comprehensive understanding of the underlying evolutionary pathways.

## Conclusion

This study provides new insights into the genetic diversity of Argentinian *Brassica rapa* within a global context. Despite their feral status, Argentinian accessions exhibit distinct genotypic profiles, likely shaped by local adaptation, highlighting their potential value as breeding material, as reported for other feral crops (Li *et al.* 2017). The lack of genetic differentiation between resistant and susceptible individuals suggests a single or limited introgression event followed by the spread of resistance within the local gene pool. Our results indicate multiple origins for Argentinian populations, including introductions from neighbouring countries for susceptible accessions and local gene flow from unreported transgenic *B. napus* cultivation leading to resistance introgression. These findings advance our understanding of *B. rapa* as a noxious weed in Argentinian agroecosystems and inform weed management strategies, while also providing a case study of transgene escape and rapid adaptation in weedy crop relatives. The distinct genetic background of Argentinian feral populations further underscores their potential as a reservoir of genetic diversity for future crop improvement.

## Supplementary Material

plag018_Supplementary_Data

## Data Availability

Raw sequencing data are available in the NCBI Sequence Read Archive (SRA) under BioProject accession PRJNA1443999, BioSample SAMN56804269. A barcode key file containing the barcode-to-sample assignment used for demultiplexing is available in Figshare (https://doi.org/10.6084/m9.figshare.31906834).
