# Supplementary material for: Unveiling the genetic diversity and ancestry of Brassica rapa weeds in Argentina: evidence for local adaptation and feralization
Source: AoB Plants. 2026 Apr 6;18(2):plag018. doi: 10.1093/aobpla/plag018 (PMC13093908; doi:10.1093/aobpla/plag018)
Supplement: plag018_Supplementary_Data [file plag018_supplementary_data.pdf]

Supporting Information

Title: Unveiling the genetic diversity and ancestry of *Brassica rapa* weeds in Argentina: Evidence for local adaptation and feralization

Authors: Sofía G. Tillería, Alejandro Presotto, Claudio Pandolfo, Alex C. McAlvay, Eve Emshwiller, Makenzie E. Mabry, Kevin A. Bird, María Soledad Ureta

Table S1. Description of the worldwide populations evaluated in this study.

Sample	*Origin*	*Taxonomy*	*Type*	*ID*
aa_BAL0802	Argentina	*Brassica rapa*	Weedy	AR_WILD_FERAL
aa_BAL0803	Argentina	*Brassica rapa*	Weedy	AR_WILD_FERAL
aa_BAL0804	Argentina	*Brassica rapa*	Weedy	AR_WILD_FERAL
aa_BAL0805	Argentina	*Brassica rapa*	Weedy	AR_WILD_FERAL
aa_BAL13A01	Argentina	*Brassica rapa*	Weedy	AR_WILD_FERAL
aa_BAL13A02	Argentina	*Brassica rapa*	Weedy	AR_WILD_FERAL
aa_BAL13A05	Argentina	*Brassica rapa*	Weedy	AR_WILD_FERAL
aa_BAL1901	Argentina	*Brassica rapa*	Weedy	AR_WILD_FERAL
aa_BAL1902	Argentina	*Brassica rapa*	Weedy	AR_WILD_FERAL
aa_BAL1903	Argentina	*Brassica rapa*	Weedy	AR_WILD_FERAL
aa_BAL1904	Argentina	*Brassica rapa*	Weedy	AR_WILD_FERAL
aa_BAL1905	Argentina	*Brassica rapa*	Weedy	AR_WILD_FERAL
aa_ERI01	Argentina	*Brassica rapa*	Weedy	AR_WILD_FERAL
aa_ERI02	Argentina	*Brassica rapa*	Weedy	AR_WILD_FERAL
aa_ERI03	Argentina	*Brassica rapa*	Weedy	AR_WILD_FERAL
aa_ERI04	Argentina	*Brassica rapa*	Weedy	AR_WILD_FERAL
aa_ERI05	Argentina	*Brassica rapa*	Weedy	AR_WILD_FERAL
aa_FAL01	Argentina	*Brassica rapa*	Weedy	AR_WILD_FERAL
aa_FAL03	Argentina	*Brassica rapa*	Weedy	AR_WILD_FERAL
aa_FAL04	Argentina	*Brassica rapa*	Weedy	AR_WILD_FERAL
aa_FAL05	Argentina	*Brassica rapa*	Weedy	AR_WILD_FERAL
aa_JUA01	Argentina	*Brassica rapa*	Weedy	AR_WILD_FERAL
aa_JUA02	Argentina	*Brassica rapa*	Weedy	AR_WILD_FERAL
aa_JUA03	Argentina	*Brassica rapa*	Weedy	AR_WILD_FERAL
aa_JUA04	Argentina	*Brassica rapa*	Weedy	AR_WILD_FERAL
aa_JUA05	Argentina	*Brassica rapa*	Weedy	AR_WILD_FERAL
aa_LDU1201	Argentina	*Brassica rapa*	Weedy	AR_WILD_FERAL
aa_LDU1203	Argentina	*Brassica rapa*	Weedy	AR_WILD_FERAL
aa_LDU1801	Argentina	*Brassica rapa*	Weedy	AR_WILD_FERAL
aa_LDU1802	Argentina	*Brassica rapa*	Weedy	AR_WILD_FERAL
aa_LDU1803	Argentina	*Brassica rapa*	Weedy	AR_WILD_FERAL
aa_LDU1804	Argentina	*Brassica rapa*	Weedy	AR_WILD_FERAL
aa_LDU1805	Argentina	*Brassica rapa*	Weedy	AR_WILD_FERAL
aa_LSA01	Argentina	*Brassica rapa*	Weedy	AR_WILD_FERAL
aa_LSA03	Argentina	*Brassica rapa*	Weedy	AR_WILD_FERAL
aa_LSA05	Argentina	*Brassica rapa*	Weedy	AR_WILD_FERAL
aa_NEC04	Argentina	*Brassica rapa*	Weedy	AR_WILD_FERAL
aa_NEC05	Argentina	*Brassica rapa*	Weedy	AR_WILD_FERAL
aa_PAN02	Argentina	*Brassica rapa*	Weedy	AR_WILD_FERAL
aa_PAN03	Argentina	*Brassica rapa*	Weedy	AR_WILD_FERAL
aa_PAN04	Argentina	*Brassica rapa*	Weedy	AR_WILD_FERAL
aa_PAN05	Argentina	*Brassica rapa*	Weedy	AR_WILD_FERAL
aa_RCU01	Argentina	*Brassica rapa*	Weedy	AR_WILD_FERAL
aa_RCU02	Argentina	*Brassica rapa*	Weedy	AR_WILD_FERAL
aa_RCU03	Argentina	*Brassica rapa*	Weedy	AR_WILD_FERAL

aa_RCU04	Argentina	*Brassica rapa*	Weedy	AR_WILD_FERAL
aa_RCU05	Argentina	*Brassica rapa*	Weedy	AR_WILD_FERAL
aa_SCB01	Argentina	*Brassica rapa*	Weedy	AR_WILD_FERAL
aa_SCB02	Argentina	*Brassica rapa*	Weedy	AR_WILD_FERAL
aa_SCB03	Argentina	*Brassica rapa*	Weedy	AR_WILD_FERAL
aa_SCB04	Argentina	*Brassica rapa*	Weedy	AR_WILD_FERAL
aa_SCB05	Argentina	*Brassica rapa*	Weedy	AR_WILD_FERAL
aa_TAFI01	Argentina	*Brassica rapa*	Weedy	AR_WILD_FERAL
aa_TAFI02	Argentina	*Brassica rapa*	Weedy	AR_WILD_FERAL
aa_TAFI03	Argentina	*Brassica rapa*	Weedy	AR_WILD_FERAL
aa_TAFI04	Argentina	*Brassica rapa*	Weedy	AR_WILD_FERAL
NGB162411Boleracea	Europa	*Brassica oleracea*	Broccoli	BROCCOLI
G 28899	China	*B.rapa subsp.Chinensis*	bok choy	C_ASIA
G 28900	China	*B.rapa subsp.Chinensis*	bok choy	C_ASIA
G 28902	China	*B.rapa subsp.Chinensis*	bok choy	C_ASIA
G 29043	China	*B.rapa subsp.Chinensis*	bok choy	C_ASIA
G 29917	China	*B.rapa subsp.Chinensis*	bok choy	C_ASIA
PI 430485	China	*B.rapa subsp.Chinensis*	bok choy	C_ASIA
PI 662540	China	*B.rapa subsp.Chinensis*	bok choy	C_ASIA
PI 662542	China	*B.rapa subsp.Chinensis*	bok choy	C_ASIA
PI 662543	China	*B.rapa subsp.Chinensis*	bok choy	C_ASIA
PI 662544	China	*B.rapa subsp.Chinensis*	bok choy	C_ASIA
PI 662545	China	*B.rapa subsp.Chinensis*	bok choy	C_ASIA
PI 662546	China	*B.rapa subsp.Chinensis*	bok choy	C_ASIA
PI 662547	China	*B.rapa subsp.Chinensis*	bok choy	C_ASIA
PI 662548	China	*B.rapa subsp.Chinensis*	bok choy	C_ASIA
PI 662549	China	*B.rapa subsp.Chinensis*	bok choy	C_ASIA
PI 662550	China	*B.rapa subsp.Chinensis*	bok choy	C_ASIA
PI 662553	China	*B.rapa subsp.Chinensis*	bok choy	C_ASIA
PI 662554	China	*B.rapa subsp.Chinensis*	bok choy	C_ASIA
PI 662555	China	*B.rapa subsp.Chinensis*	bok choy	C_ASIA
PI 478323	China	*B.rapa subsp.Chinensis*	bok choy	C_ASIA
PI 662541	China	*B.rapa subsp.Chinensis*	bok choy	C_ASIA
PI 662557	China	*B.rapa subsp.Chinensis*	bok choy	C_ASIA
PI 662681	Japan	*B.rapa subsp.Chinensis*	bok choy	C_ASIA
PI 662679	Japan	*B.rapa subsp.Chinensis*	bok choy	C_ASIA
PI 662558	China	*B.rapa subsp.Chinensis*	bok choy	C_ASIA
PI 662551	China	*B.rapa subsp.Chinensis*	bok choy	C_ASIA
PI 662552	China	*B.rapa subsp.Chinensis*	bok choy	C_ASIA
G 30708	China	*B.rapa subsp.Chinensis*	bok choy	C_ASIA
G 30955	China	*B.rapa subsp.Chinensis*	bok choy	C_ASIA
G 30956	China	*B.rapa subsp.Chinensis*	bok choy	C_ASIA
G 31776	China	*B.rapa subsp.Chinensis*	bok choy	C_ASIA
G 31777	China	*B.rapa subsp.Chinensis*	bok choy	C_ASIA
G 31784	China	*B.rapa subsp.Chinensis*	bok choy	C_ASIA
G 31785	China	*B.rapa subsp.Chinensis*	bok choy	C_ASIA
G 32371	China	*B.rapa subsp.Chinensis*	bok choy	C_ASIA
PI 257239	China	*B.rapa subsp.Chinensis*	bok choy	C_ASIA
PI 430484	China	*B.rapa subsp.Chinensis*	bok choy	C_ASIA
PI 430486	China	*B.rapa subsp.Chinensis*	bok choy	C_ASIA
PI 478324	China	*B.rapa subsp.Chinensis*	bok choy	C_ASIA
PI 633165	China	*B.rapa subsp.Chinensis*	bok choy	C_ASIA
PI 662724	China	*B.rapa subsp.Chinensis*	bok choy	C_ASIA
PI 662725	China	*B.rapa subsp.Chinensis*	bok choy	C_ASIA
PI 662726	China	*B.rapa subsp.Chinensis*	bok choy	C_ASIA
PI 662727	China	*B.rapa subsp.Chinensis*	bok choy	C_ASIA
PI 662728	China	*B.rapa subsp.Chinensis*	bok choy	C_ASIA
PI 662739	China	*B.rapa subsp.Chinensis*	bok choy	C_ASIA
PI 662740	China	*B.rapa subsp.Chinensis*	bok choy	C_ASIA
PI 662751	China	*B.rapa subsp.Chinensis*	bok choy	C_ASIA

PI 662752	China	*B.rapa subsp.Chinensis*	bok choy	C_ASIA
PI 662753	China	*B.rapa subsp.Chinensis*	bok choy	C_ASIA
PI 662754	China	*B.rapa subsp.Chinensis*	bok choy	C_ASIA
G 30792	Japan	*B.rapa subsp.Chinensis*	bok choy	C_ASIA
G 30794	Japan	*B.rapa subsp.Chinensis*	bok choy	C_ASIA
G 32370	Japan	*B.rapa subsp.Chinensis*	bok choy	C_ASIA
PI 662678	Japan	*B.rapa subsp.Chinensis*	bok choy	C_ASIA
PI 662677	Japan	*B.rapa subsp.Chinensis*	bok choy	C_ASIA
PI 662680	Japan	*B.rapa subsp.Chinensis*	bok choy	C_ASIA
PI 508408	South Korea	*B.rapa subsp.Chinensis*	bok choy	C_ASIA
PI 508409	South Korea	*B.rapa subsp.Chinensis*	bok choy	C_ASIA
PI 662757	China	*B.rapa subsp.Chinensis*	bok choy	C_ASIA
PI 662758	China	*B.rapa subsp.Chinensis*	bok choy	C_ASIA
PI 662755	China	*B.rapa subsp.Chinensis*	bok choy	C_ASIA
PI 662756	China	*B.rapa subsp.Chinensis*	bok choy	C_ASIA
PI 662682	Taiwan	*B.rapa subsp.Chinensis*	bok choy	C_ASIA
PI 662556	China	*B.rapa subsp.Chinensis*	bok choy	C_ASIA
PI 340208	India	*B.rapa subsp.Dichotoma*	brown sarson	D_ASIA
PI 347594	India	*B.rapa subsp.Dichotoma*	brown sarson	D_ASIA
PI 347602	India	*B.rapa subsp.Dichotoma*	brown sarson	D_ASIA
PI 649168	India	*B.rapa subsp.Dichotoma*	brown sarson	D_ASIA
PI 649171	India	*B.rapa subsp.Dichotoma*	brown sarson	D_ASIA
PI 649173	India	*B.rapa subsp.Dichotoma*	brown sarson	D_ASIA
PI 649178	India	*B.rapa subsp.Dichotoma*	brown sarson	D_ASIA
PI 662642	India	*B.rapa subsp.Dichotoma*	brown sarson	D_ASIA
PI 633166	Nepal	*B.rapa subsp.Dichotoma*	brown sarson	D_ASIA
PI 649181	U.S.	*B.rapa subsp.Dichotoma*	brown sarson	D_NORTH_AMERICA
M143grelo	España	*B. rapa subsp. sylvestris var. esculenta*	Grelos	GRELOS
M163grelo	España	*B. rapa subsp. sylvestris var. esculenta*	Grelos	GRELOS
M173grelo	España	*subsp. sylvestris var. esculenta*	Grelos	GRELOS
M190grelo	España	*B. rapa subsp. sylvestris var. esculenta*	Grelos	GRELOS
M197grelo	España	*B. rapa subsp. sylvestris var. esculenta*	Grelos	GRELOS
M316grelo	España	*subsp. sylvestris var. esculenta*	Grelos	GRELOS
M417grelo	España	*B. rapa subsp. sylvestris var. esculenta*	Grelos	GRELOS
M472grelo	España	*B. rapa subsp. sylvestris var. esculenta*	Grelos	GRELOS
M588grelo	España	*B. rapa subsp. sylvestris var. esculenta*	Grelos	GRELOS
M599grelo	España	*subsp. sylvestris var. esculenta*	Grelos	GRELOS
M82grelo	España	*B. rapa subsp. sylvestris var. esculenta*	Grelos	GRELOS
acm25	Portugal	*Brassica rapa*	Grelos	GRELOS
acm18	Portugal	*Brassica rapa*	Turnip/grelos	GRELOS
PI 662691	Japan	*B.rapa subsp.Narinosa*	tatsoi	NAR_ASIA
PI 662690	Japan	*B.rapa subsp.Narinosa*	tatsoi	NAR_ASIA
PI 662687	Taiwan	*B.rapa subsp.Nipposinica*	mizuna	NIP_ASIA
PI 662626	Japan	*B.rapa subsp.Nipposinica*	mizuna	NIP_ASIA
PI 662689	Japan	*B.rapa subsp.Nipposinica*	mizuna	NIP_ASIA
PI 597831	Egypt	*B.rapa subsp.Oleifera*	oilseed rape	O_AFRICA
CR2212Chinaolei	China	*B. rapa ssp. oleifera*	Oilseed	O_ASIA
PI 430488	China	*B.rapa subsp.Oleifera*	oilseed rape	O_ASIA
G 30623	India	*B.rapa subsp.Oleifera*	oilseed rape	O_ASIA
BRA3039ladakh	South Asia	*B. rapa ssp. trilocularis*	Oilseed	O_ASIA
CR2672Yemen	Yemen	*B. rapa ssp. trilocularis*	Oilseed	O_ASIA
acm13	Germany	*Oilseed*	oilseed	O_EUROPE
PI 649194	Germany	*B.rapa subsp.Oleifera*	oilseed rape	O_EUROPE
acm10	Belgium	*Brassica rapa*	oilseed	O_EUROPE
acm06	Finland	*Oilseed*	oilseed	O_EUROPE
acm11	Netherlands	*Oilseed*	oilseed	O_EUROPE
acm15	Poland	*Oilseed*	oilseed	O_EUROPE
G 30238	España	*B.rapa subsp.Oleifera*	oilseed rape	O_EUROPE
PI 633168	Italy	*B.rapa subsp.Oleifera*	oilseed rape	O_EUROPE
PI 662794	Italy	*B.rapa subsp.Oleifera*	oilseed rape	O_EUROPE

PI 633169	Cuba	*B.rapa subsp.Oleifera*	oilseed rape	O_SOUTH AMERICA
G 30496	China	*B.rapa subsp.Pekinensis*	Chinese cabbage	P_ASIA
G 30688	China	*B.rapa subsp.Pekinensis*	Chinese cabbage	P_ASIA
G 30692	China	*B.rapa subsp.Pekinensis*	Chinese cabbage	P_ASIA
G 30694	China	*B.rapa subsp.Pekinensis*	Chinese cabbage	P_ASIA
G 30695	China	*B.rapa subsp.Pekinensis*	Chinese cabbage	P_ASIA
G 30697	China	*B.rapa subsp.Pekinensis*	Chinese cabbage	P_ASIA
G 30703	China	*B.rapa subsp.Pekinensis*	Chinese cabbage	P_ASIA
G 30952	China	*B.rapa subsp.Pekinensis*	Chinese cabbage	P_ASIA
G 31753	China	*B.rapa subsp.Pekinensis*	Chinese cabbage	P_ASIA
G 31759	China	*B.rapa subsp.Pekinensis*	Chinese cabbage	P_ASIA
G 31761	China	*B.rapa subsp.Pekinensis*	Chinese cabbage	P_ASIA
G 31762	China	*B.rapa subsp.Pekinensis*	Chinese cabbage	P_ASIA
G 31765	China	*B.rapa subsp.Pekinensis*	Chinese cabbage	P_ASIA
G 31773	China	*B.rapa subsp.Pekinensis*	Chinese cabbage	P_ASIA
PI 269436	China	*B.rapa subsp.Pekinensis*	Chinese cabbage	P_ASIA
PI 269437	China	*B.rapa subsp.Pekinensis*	Chinese cabbage	P_ASIA
PI 391558	China	*B.rapa subsp.Pekinensis*	Chinese cabbage	P_ASIA
PI 391559	China	*B.rapa subsp.Pekinensis*	Chinese cabbage	P_ASIA
PI 391560	China	*B.rapa subsp.Pekinensis*	Chinese cabbage	P_ASIA
PI 418957	China	*B.rapa subsp.Pekinensis*	Chinese cabbage	P_ASIA
PI 418958	China	*B.rapa subsp.Pekinensis*	Chinese cabbage	P_ASIA
PI 418959	China	*B.rapa subsp.Pekinensis*	Chinese cabbage	P_ASIA
PI 418984	China	*B.rapa subsp.Pekinensis*	Chinese cabbage	P_ASIA
PI 418985	China	*B.rapa subsp.Pekinensis*	Chinese cabbage	P_ASIA
PI 418986	China	*B.rapa subsp.Pekinensis*	Chinese cabbage	P_ASIA
PI 418987	China	*B.rapa subsp.Pekinensis*	Chinese cabbage	P_ASIA
PI 418988	China	*B.rapa subsp.Pekinensis*	Chinese cabbage	P_ASIA
PI 419007	China	*B.rapa subsp.Pekinensis*	Chinese cabbage	P_ASIA
PI 419024	China	*B.rapa subsp.Pekinensis*	Chinese cabbage	P_ASIA
PI 419025	China	*B.rapa subsp.Pekinensis*	Chinese cabbage	P_ASIA
PI 419026	China	*B.rapa subsp.Pekinensis*	Chinese cabbage	P_ASIA
PI 419027	China	*B.rapa subsp.Pekinensis*	Chinese cabbage	P_ASIA
PI 419028	China	*B.rapa subsp.Pekinensis*	Chinese cabbage	P_ASIA
PI 419038	China	*B.rapa subsp.Pekinensis*	Chinese cabbage	P_ASIA
PI 419069	China	*B.rapa subsp.Pekinensis*	Chinese cabbage	P_ASIA
PI 419070	China	*B.rapa subsp.Pekinensis*	Chinese cabbage	P_ASIA
PI 419072	China	*B.rapa subsp.Pekinensis*	Chinese cabbage	P_ASIA
PI 419073	China	*B.rapa subsp.Pekinensis*	Chinese cabbage	P_ASIA
PI 419074	China	*B.rapa subsp.Pekinensis*	Chinese cabbage	P_ASIA
PI 419105	China	*B.rapa subsp.Pekinensis*	Chinese cabbage	P_ASIA
PI 419106	China	*B.rapa subsp.Pekinensis*	Chinese cabbage	P_ASIA
PI 419127	China	*B.rapa subsp.Pekinensis*	Chinese cabbage	P_ASIA
PI 419129	China	*B.rapa subsp.Pekinensis*	Chinese cabbage	P_ASIA
PI 419130	China	*B.rapa subsp.Pekinensis*	Chinese cabbage	P_ASIA
PI 419131	China	*B.rapa subsp.Pekinensis*	Chinese cabbage	P_ASIA
PI 427091	China	*B.rapa subsp.Pekinensis*	Chinese cabbage	P_ASIA
PI 427094	China	*B.rapa subsp.Pekinensis*	Chinese cabbage	P_ASIA
PI 430583	China	*B.rapa subsp.Pekinensis*	Chinese cabbage	P_ASIA
PI 430610	China	*B.rapa subsp.Pekinensis*	Chinese cabbage	P_ASIA
PI 432771	China	*B.rapa subsp.Pekinensis*	Chinese cabbage	P_ASIA
PI 432774	China	*B.rapa subsp.Pekinensis*	Chinese cabbage	P_ASIA
PI 432776	China	*B.rapa subsp.Pekinensis*	Chinese cabbage	P_ASIA
PI 432777	China	*B.rapa subsp.Pekinensis*	Chinese cabbage	P_ASIA
PI 432779	China	*B.rapa subsp.Pekinensis*	Chinese cabbage	P_ASIA
PI 436668	China	*B.rapa subsp.Pekinensis*	Chinese cabbage	P_ASIA
PI 436669	China	*B.rapa subsp.Pekinensis*	Chinese cabbage	P_ASIA
PI 436670	China	*B.rapa subsp.Pekinensis*	Chinese cabbage	P_ASIA
PI 436671	China	*B.rapa subsp.Pekinensis*	Chinese cabbage	P_ASIA
PI 478342	China	*B.rapa subsp.Pekinensis*	Chinese cabbage	P_ASIA

PI 478345	China	*B.rapa subsp.Pekinensis*	Chinese cabbage	P_ASIA
PI 478348	China	*B.rapa subsp.Pekinensis*	Chinese cabbage	P_ASIA
PI 478349	China	*B.rapa subsp.Pekinensis*	Chinese cabbage	P_ASIA
PI 487420	China	*B.rapa subsp.Pekinensis*	Chinese cabbage	P_ASIA
PI 487421	China	*B.rapa subsp.Pekinensis*	Chinese cabbage	P_ASIA
PI 487422	China	*B.rapa subsp.Pekinensis*	Chinese cabbage	P_ASIA
PI 489751	China	*B.rapa subsp.Pekinensis*	Chinese cabbage	P_ASIA
PI 518839	China	*B.rapa subsp.Pekinensis*	Chinese cabbage	P_ASIA
PI 518840	China	*B.rapa subsp.Pekinensis*	Chinese cabbage	P_ASIA
PI 518841	China	*B.rapa subsp.Pekinensis*	Chinese cabbage	P_ASIA
PI 518842	China	*B.rapa subsp.Pekinensis*	Chinese cabbage	P_ASIA
PI 518843	China	*B.rapa subsp.Pekinensis*	Chinese cabbage	P_ASIA
PI 518844	China	*B.rapa subsp.Pekinensis*	Chinese cabbage	P_ASIA
PI 518845	China	*B.rapa subsp.Pekinensis*	Chinese cabbage	P_ASIA
PI 518846	China	*B.rapa subsp.Pekinensis*	Chinese cabbage	P_ASIA
PI 518847	China	*B.rapa subsp.Pekinensis*	Chinese cabbage	P_ASIA
PI 527322	China	*B.rapa subsp.Pekinensis*	Chinese cabbage	P_ASIA
PI 527323	China	*B.rapa subsp.Pekinensis*	Chinese cabbage	P_ASIA
PI 590995	China	*B.rapa subsp.Pekinensis*	Chinese cabbage	P_ASIA
PI 662572	China	*B.rapa subsp.Pekinensis*	Chinese cabbage	P_ASIA
PI 662636	China	*B.rapa subsp.Pekinensis*	Chinese cabbage	P_ASIA
PI 662637	China	*B.rapa subsp.Pekinensis*	Chinese cabbage	P_ASIA
PI 662638	China	*B.rapa subsp.Pekinensis*	Chinese cabbage	P_ASIA
PI 662639	China	*B.rapa subsp.Pekinensis*	Chinese cabbage	P_ASIA
PI 662640	China	*B.rapa subsp.Pekinensis*	Chinese cabbage	P_ASIA
PI 662653	China	*B.rapa subsp.Pekinensis*	Chinese cabbage	P_ASIA
PI 662655	China	*B.rapa subsp.Pekinensis*	Chinese cabbage	P_ASIA
PI 662656	China	*B.rapa subsp.Pekinensis*	Chinese cabbage	P_ASIA
PI 662657	China	*B.rapa subsp.Pekinensis*	Chinese cabbage	P_ASIA
PI 662658	China	*B.rapa subsp.Pekinensis*	Chinese cabbage	P_ASIA
PI 662717	China	*B.rapa subsp.Pekinensis*	Chinese cabbage	P_ASIA
PI 662718	China	*B.rapa subsp.Pekinensis*	Chinese cabbage	P_ASIA
PI 662719	China	*B.rapa subsp.Pekinensis*	Chinese cabbage	P_ASIA
PI 662720	China	*B.rapa subsp.Pekinensis*	Chinese cabbage	P_ASIA
PI 662721	China	*B.rapa subsp.Pekinensis*	Chinese cabbage	P_ASIA
PI 279857	Japan	*B.rapa subsp.Pekinensis*	Chinese cabbage	P_ASIA
PI 662676	Japan	*B.rapa subsp.Pekinensis*	Chinese cabbage	P_ASIA
PI 508414	South Korea	*B.rapa subsp.Pekinensis*	Chinese cabbage	P_ASIA
PI 508416	South Korea	*B.rapa subsp.Pekinensis*	Chinese cabbage	P_ASIA
PI 508418	South Korea	*B.rapa subsp.Pekinensis*	Chinese cabbage	P_ASIA
PI 508420	South Korea	*B.rapa subsp.Pekinensis*	Chinese cabbage	P_ASIA
PI 508421	South Korea	*B.rapa subsp.Pekinensis*	Chinese cabbage	P_ASIA
PI 508422	South Korea	*B.rapa subsp.Pekinensis*	Chinese cabbage	P_ASIA
PI 508423	South Korea	*B.rapa subsp.Pekinensis*	Chinese cabbage	P_ASIA
PI 508424	South Korea	*B.rapa subsp.Pekinensis*	Chinese cabbage	P_ASIA
PI 508425	South Korea	*B.rapa subsp.Pekinensis*	Chinese cabbage	P_ASIA
PI 508426	South Korea	*B.rapa subsp.Pekinensis*	Chinese cabbage	P_ASIA
PI 508427	South Korea	*B.rapa subsp.Pekinensis*	Chinese cabbage	P_ASIA
PI 508428	South Korea	*B.rapa subsp.Pekinensis*	Chinese cabbage	P_ASIA
PI 508429	South Korea	*B.rapa subsp.Pekinensis*	Chinese cabbage	P_ASIA
PI 508430	South Korea	*B.rapa subsp.Pekinensis*	Chinese cabbage	P_ASIA
PI 257236	Thailand	*B.rapa subsp.Pekinensis*	Chinese cabbage	P_ASIA
PI 257238	Thailand	*B.rapa subsp.Pekinensis*	Chinese cabbage	P_ASIA
PI 508415	South Korea	*B.rapa subsp.Pekinensis*	Chinese cabbage	P_ASIA
PI 662675	China	*B.rapa subsp.Pekinensis*	bok choy	P_ASIA
PI 662722	China	*B.rapa subsp.Pekinensis*	Chinese cabbage	P_ASIA
PI 662723	China	*B.rapa subsp.Pekinensis*	Chinese cabbage	P_ASIA
PI 662738	China	*B.rapa subsp.Pekinensis*	Chinese cabbage	P_ASIA
PI 662741	China	*B.rapa subsp.Pekinensis*	Chinese cabbage	P_ASIA
PI 662742	China	*B.rapa subsp.Pekinensis*	Chinese cabbage	P_ASIA

PI 662743	China	*B.rapa subsp.Pekinensis*	Chinese cabbage	P_ASIA
PI 662744	China	*B.rapa subsp.Pekinensis*	Chinese cabbage	P_ASIA
PI 662745	China	*B.rapa subsp.Pekinensis*	Chinese cabbage	P_ASIA
PI 662746	China	*B.rapa subsp.Pekinensis*	Chinese cabbage	P_ASIA
PI 662747	China	*B.rapa subsp.Pekinensis*	Chinese cabbage	P_ASIA
PI 662748	China	*B.rapa subsp.Pekinensis*	Chinese cabbage	P_ASIA
PI 662749	China	*B.rapa subsp.Pekinensis*	Chinese cabbage	P_ASIA
PI 662750	China	*B.rapa subsp.Pekineniss*	Chinese cabbage	P_ASIA
PI 662791	China	*B.rapa subsp.Pekinensis*	Chinese cabbage	P_ASIA
PI 508417	South Korea	*B.rapa subsp.Pekinensis*	Chinese cabbage	P_ASIA
PI 269438	Pakistan	*B.rapa subsp.Pekinensis*	Chinese cabbage	P_ASIA
PI 257237	Thailand	*B.rapa subsp.Pekinensis*	Chinese cabbage	P_ASIA
PI 662562	Netherlands	*B.rapa subsp.Pekinensis*	Chinese cabbage	P_EUROPE
G 30441	U.S.	*B.rapa subsp.Pekinensis*	Chinese cabbage	P_NORTH_AMERICA
G 30784	U.S.	*B.rapa subsp.Pekinensis*	Chinese cabbage	P_NORTH_AMERICA
G 30787	U.S.	*B.rapa subsp.Pekinensis*	Chinese cabbage	P_NORTH_AMERICA
PI 644006	U.S.	*B.rapa subsp.Pekinensis*	Chinese cabbage	P_NORTH_AMERICA
PI 644007	U.S.	*B.rapa subsp.Pekinensis*	Chinese cabbage	P_NORTH_AMERICA
PI 662611	U.S.	*B.rapa subsp.Pekinensis*	Chinese cabbage	P_NORTH_AMERICA
PI 662612	U.S.	*B.rapa subsp.Pekinensis*	Chinese cabbage	P_NORTH_AMERICA
PI 662613	U.S.	*B.rapa subsp.Pekinensis*	Chinese cabbage	P_NORTH_AMERICA
PI 662614	U.S.	*B.rapa subsp.Pekinensis*	Chinese cabbage	P_NORTH_AMERICA
PI 662615	U.S.	*B.rapa subsp.Pekinensis*	Chinese cabbage	P_NORTH_AMERICA
PI 662617	U.S.	*B.rapa subsp.Pekinensis*	Chinese cabbage	P_NORTH_AMERICA
PI 662618	U.S.	*B.rapa subsp.Pekinensis*	Chinese cabbage	P_NORTH_AMERICA
PI 662619	U.S.	*B.rapa subsp.Pekinensis*	Chinese cabbage	P_NORTH_AMERICA
PI 662620	U.S.	*B.rapa subsp.Pekinensis*	Chinese cabbage	P_NORTH_AMERICA
PI 662621	U.S.	*B.rapa subsp.Pekinensis*	Chinese cabbage	P_NORTH_AMERICA
PI 234600	Australia	*B.rapa subsp.Pekinensis*	Chinese cabbage	P_OCEANIA
G 31344	China	*B.rapa subsp.Parachinensis*	choy sum	PARA_ASIA
PI 390963	Hong Kong	*B.rapa subsp.Parachinensis*	choy sum	PARA_ASIA
PI 390964	Hong Kong	*B.rapa subsp.Parachinensis*	choy sum	PARA_ASIA
PI 662783	Indonesia	*B.rapa subsp.Parachinensis,*	choy sum	PARA_ASIA
PI 662616	U.S.	*B.rapa subsp.perviridis*	komatsuna	PER_NORTH_AMERICA
PI 662627	U.S.	*B.rapa subsp.perviridis*	komatsuna	PER_NORTH_AMERICA
bavinccentoventesta	Europa	*B. rapa ssp. sylvestris var. esculenta*	Rapini	RAPINI
bavincnoventina	Europa	*B. rapa ssp. sylvestris var. esculenta*	Rapini	RAPINI
bavincricamrzno	Europa	*B. rapa ssp. sylvestris var. esculenta*	Rapini	RAPINI
CR2551italian	Italy	*B. rapa ssp. sylvestris var. esculenta*	Rapini	RAPINI
PI6331680Italy	Italy	*B. rapa ssp. sylvestris var. esculenta*	Rapini	RAPINI
acm19	Egypt	*Brassica rapa*	Turnip	T_AFRICA
acm20	Egypt		Turnip	T_AFRICA
acm21	Egypt		Turnip	T_AFRICA
PI 250004	Egypt	*Brassica rapa*	turnip	T_AFRICA
BRA1718TLibya	Libia	*B. rapa ssp. rapa*	turnip	T_AFRICA
CR2552OLibya	Libia	*B. rapa ssp. rapa*	Turnip	T_AFRICA
PI 426249	Pakistan	*Brassica rapa*	turnip/turnip rape?	T_ASIA
PI 603023	Pakistan	*Brassica rapa*	turnip?	T_ASIA
PI125797TAfg	Afghanistan	*B. rapa ssp. rapa*	Turnip	T_ASIA
PI125798TAfg	Afghanistan	*B. rapa ssp. rapa*	Turnip	T_ASIA
PI127440TAfg	Afghanistan	*B. rapa ssp. rapa*	Turnip	T_ASIA
PI211582TAfg	Afghanistan	*B. rapa ssp. rapa*	Turnip	T_ASIA
PI268368TAfg	Afghanistan	*B. rapa ssp. rapa*	Turnip	T_ASIA
acm08	Bhutan	*Brassica rapa*	Turnip	T_ASIA
acm12	Bhutan	*Brassica rapa*	Turnip	T_ASIA
acm16	Bhutan	*Brassica rapa*	Turnip	T_ASIA
acm17	Bhutan	*Brassica rapa*	Turnip	T_ASIA
PI 179863	India	*Brassica rapa*	turnip?	T_ASIA
PI222236TIran	Iran	*B. rapa ssp. rapa*	Turnip	T_ASIA
BRA1901TIraq	Iraq	*B. rapa ssp. rapa*	Turnip	T_ASIA

acm23	Japan		Turnip	T_ASIA
PI 662559	Japan	*B.rapa subsp.rapa*	turnip	T_ASIA
PI 662694	Japan	*B.rapa subsp.rapa*	turnip	T_ASIA
PI269439TPak	Pakistan	*B. rapa ssp. rapa*	Turnip	T_ASIA
PI269441TPak	Pakistan	*B. rapa ssp. rapa*	Turnip	T_ASIA
PI269442TPak	Pakistan	*B. rapa ssp. rapa*	Turnip	T_ASIA
acm24	Syria	*Brassica rapa*	Turnip	T_ASIA
acm26	Syria	*Brassica rapa*	Turnip	T_ASIA
acm27	Syria	*Brassica rapa*	Turnip	T_ASIA
BRA1717TajikT	Tajikistan	*B. rapa ssp. rapa*	turnip	T_ASIA
BRA2985Tajik	Tajikistan	*B. rapa ssp. rapa*	Turnip	T_ASIA
BRA2196Turkey	Turkey	*B. rapa ssp. rapa*	turnip	T_ASIA
PI169061TTur	Turkey	*B. rapa ssp. rapa*	turnip	T_ASIA
PI169064balikesir	Turkey	*B. rapa ssp. rapa*	turnip	T_ASIA
PI169070TTur	Turkey	*B. rapa ssp. rapa*	turnip	T_ASIA
PI177286hakkari	Turkey	*B. rapa ssp. rapa*	turnip	T_ASIA
PI183664TTur	Turkey	*B. rapa ssp. rapa*	Turnip	T_ASIA
PI649185OGermany	Germany	*B. rapa ssp. rapa*	Turnip	T_EUROPE
G 31841	Germany	*B.rapa subsp.rapa*	turnip	T_EUROPE
BRA1018TAustria	Austria	*B. rapa ssp. rapa*	Turnip	T_EUROPE
PI649189Belgium	Belgium	*B. rapa ssp. rapa*	Turnip	T_EUROPE
PI633178TDenmark	Denmark	*B. rapa ssp. rapa*	Turnip	T_EUROPE
BR50226valenc	España	*B. rapa ssp. rapa*	turnip	T_EUROPE
BR50227cuenc	España	*B. rapa ssp. rapa*	Spanish turnip	T_EUROPE
BRS0225Valenc	España	*B. rapa ssp. rapa*	Turnip	T_EUROPE
EXTBRS0228TCastellon	España	*B. rapa ssp. rapa*	Turnip	T_EUROPE
EXTBRSO231Caceres	España	*B. rapa ssp. rapa*	Turnip	T_EUROPE
EXTBRSO239Santander	España	*B. rapa ssp. rapa*	turnip	T_EUROPE
BRA1709TTunisian	Europa	*B. rapa ssp. rapa*	turnip	T_EUROPE
BRA1892TGeorgia	Europa	*B. rapa ssp. rapa*	turnip	T_EUROPE
questionFinlandBRA1814	Europa	*B. rapa ssp. rapa*	Turnip	T_EUROPE
questionYugoBRA2780	Europa	*B. rapa ssp. rapa*	Turnip	T_EUROPE
Rejogochib9	Europa	*B. rapa ssp. rapa*	Turnip	T_EUROPE
PI 662695	France	*B.rapa subsp.rapa*	turnip	T_EUROPE
PI 662795	Netherlands	*B.rapa subsp.rapa*	turnip	T_EUROPE
BRA917THungary	Hungria	*B. rapa ssp. rapa*	turnip	T_EUROPE
BRA1831TItaly	Italy	*B. rapa ssp. rapa*	turnip	T_EUROPE
BRA1894TItaly	Italy	*B. rapa ssp. rapa*	Turnip	T_EUROPE
BRA2731TItaly	Italy	*B. rapa ssp. rapa*	turnip	T_EUROPE
PI 662686	Italy	*B.rapa subsp.rapa*	turnip	T_EUROPE
CR1542OPoland	Poland	*B. rapa ssp. rapa*	turnip	T_EUROPE
acm22	Portugal	*Brassica rapa*	Turnip	T_EUROPE
G 30479	U.S.	*B.rapa subsp.rapa*	turnip	T_NORTH_AMERICA
G 30808	U.S.	*B.rapa subsp.rapa*	turnip	T_NORTH_AMERICA
PI 662628	U.S.	*B.rapa subsp.rapa*	turnip	T_NORTH_AMERICA
PI 662629	U.S.	*B.rapa subsp.rapa*	turnip	T_NORTH_AMERICA
PI 662630	U.S.	*B.rapa subsp.rapa*	turnip	T_NORTH_AMERICA
PI 662631	U.S.	*B.rapa subsp.rapa*	turnip	T_NORTH_AMERICA
PI 662632	U.S.	*B.rapa subsp.rapa*	turnip	T_NORTH_AMERICA
PI 662693	U.S.	*B.rapa subsp.rapa*	turnip	T_NORTH_AMERICA
PI 662716	U.S.	*B.rapa subsp.rapa*	turnip	T_NORTH_AMERICA
PI 426234	Pakistan	*Brassica rapa*	toria	TOR_ASIA
PI 426236	Pakistan	*Brassica rapa*	toria	TOR_ASIA
PI 426247	Pakistan	*Brassica rapa*	toria	TOR_ASIA
PI 426248	Pakistan	*Brassica rapa*	toria	TOR_ASIA
PI 426252	Pakistan	*Brassica rapa*	toria	TOR_ASIA
acm04	Algeria	*Brassica rapa*	wild/feral	WF_AFRICA
acm09	Algeria	*Brassica rapa*	wild/feral	WF_AFRICA
acm01	Japan	*Brassica rapa*	wild/feral	WF_ASIA
CR2234Russiasilv	Rusia	*B. rapa ssp. sylvestris*	Spontaneous	WF_ASIA

acm14	Turkey	*Brassica rapa*	wild/feral	WF_ASIA
CR2269senzatesta		*B. rapa ssp. sylvestris*	Spontaneous	WF_CAUCASO
BRA2809Turk	Turkey	*B. rapa ssp. sylvestris*	Spontaneous	WF_CAUCASO
Cr2211geor	Georgia	*B. rapa ssp. sylvestris*	Spontaneous	WF_CAUCASO
CR2241georg	Georgia	*B. rapa ssp. sylvestris*	Spontaneous	WF_CAUCASO
CR2354georg	Georgia	*B. rapa ssp. sylvestris*	Spontaneous	WF_CAUCASO
Georgiahoney	Georgia	*B. rapa ssp. sylvestris*	Spontaneous	WF_CAUCASO
CR2213austria	Austria	*B. rapa ssp. sylvestris*	Spontaneous	WF_EUROPE
CR1538OSlovakia	Slovakia	*B. rapa ssp. sylvestris*	Spontaneous	WF_EUROPE
CR1578slovak	Slovakia	*B. rapa ssp. sylvestris*	Spontaneous	WF_EUROPE
CR2210slovak	Slovakia	*B. rapa ssp. sylvestris*	Spontaneous	WF_EUROPE
Ewijk	Europa	*B. rapa ssp. sylvestris*	Spontaneous	WF_EUROPE
GaliniMaarsen	Europa	*B. rapa ssp. sylvestris*	Spontaneous	WF_EUROPE
Lindbjerg	Europa	*B. rapa ssp. sylvestris*	Spontaneous	WF_EUROPE
Lonstorp	Europa	*B. rapa ssp. sylvestris*	Spontaneous	WF_EUROPE
NGB1320661Horne	Europa	*B. rapa ssp. sylvestris*	Spontaneous	WF_EUROPE
NGB132068.1WOutrup	Europa	*B. rapa ssp. sylvestris*	Spontaneous	WF_EUROPE
Wageningen	Europa	*B. rapa ssp. sylvestris*	Spontaneous	WF_EUROPE
acm02	Italy	*Brassica rapa*	wild/feral	WF_EUROPE
PI537003Serb	Serbia	*B. rapa ssp. sylvestris*	Spontaneous	WF_EUROPE
BRA2465sweden	Sweden	*B. rapa ssp. sylvestris*	Spontaneous	WF_EUROPE
acm03	UK	*Brassica rapa*	wild/feral	WF_EUROPE
CR2355Britain	UK	*B. rapa ssp. sylvestris*	Spontaneous	WF_EUROPE
14Alej46a	Mexico	*B. rapa ssp. sylvestris*	Spontaneous	WF_MEXICO
14Alej46c	Mexico	*B. rapa ssp. sylvestris*	Spontaneous	WF_MEXICO
14Alej47	Mexico	*B. rapa ssp. sylvestris*	Spontaneous	WF_MEXICO
14Alej62	Mexico	*B. rapa ssp. sylvestris*	Spontaneous	WF_MEXICO
14DFmark11b	Mexico	*B. rapa ssp. sylvestris*	Spontaneous	WF_MEXICO
14Pan59	Mexico	*B. rapa ssp. sylvestris*	Spontaneous	WF_MEXICO
14Pan61b	Mexico	*B. rapa ssp. sylvestris*	Spontaneous	WF_MEXICO
2Colpos1	Mexico	*B. rapa ssp. sylvestris*	Spontaneous	WF_MEXICO
3Colpos2	Mexico	*B. rapa ssp. sylvestris*	Spontaneous	WF_MEXICO
3Colpos3	Mexico	*B. rapa ssp. sylvestris*	Spontaneous	WF_MEXICO
AboveChuneloatfield4	Mexico	*B. rapa ssp. sylvestris*	Spontaneous	WF_MEXICO
AboveChuneloatfield5	Mexico	*B. rapa ssp. sylvestris*	Spontaneous	WF_MEXICO
AboveChuneloatfield6	Mexico	*B. rapa ssp. sylvestris*	Spontaneous	WF_MEXICO
AboveChuneloatfield7	Mexico	*B. rapa ssp. sylvestris*	Spontaneous	WF_MEXICO
Alejf1	Mexico	*B. rapa ssp. sylvestris*	Spontaneous	WF_MEXICO
Alejf2	Mexico	*B. rapa ssp. sylvestris*	Spontaneous	WF_MEXICO
Alejf3	Mexico	*B. rapa ssp. sylvestris*	Spontaneous	WF_MEXICO
Alejf4	Mexico	*B. rapa ssp. sylvestris*	Spontaneous	WF_MEXICO
Alejf5	Mexico	*B. rapa ssp. sylvestris*	Spontaneous	WF_MEXICO
Analco1	Mexico	*B. rapa ssp. sylvestris*	Spontaneous	WF_MEXICO
Analco2	Mexico	*B. rapa ssp. sylvestris*	Spontaneous	WF_MEXICO
Analco3	Mexico	*B. rapa ssp. sylvestris*	Spontaneous	WF_MEXICO
Analco4	Mexico	*B. rapa ssp. sylvestris*	Spontaneous	WF_MEXICO
Analco5	Mexico	*B. rapa ssp. sylvestris*	Spontaneous	WF_MEXICO
Analco6	Mexico	*B. rapa ssp. sylvestris*	Spontaneous	WF_MEXICO
Analco7	Mexico	*B. rapa ssp. sylvestris*	Spontaneous	WF_MEXICO
Analco8	Mexico	*B. rapa ssp. sylvestris*	Spontaneous	WF_MEXICO
Ang8	Mexico	*B. rapa ssp. sylvestris*	Spontaneous	WF_MEXICO
Berta1	Mexico	*B. rapa ssp. sylvestris*	Spontaneous	WF_MEXICO
Berta2	Mexico	*B. rapa ssp. sylvestris*	Spontaneous	WF_MEXICO
Bneigh1	Mexico	*B. rapa ssp. sylvestris*	Spontaneous	WF_MEXICO
Bneigh2	Mexico	*B. rapa ssp. sylvestris*	Spontaneous	WF_MEXICO
Bneighc	Mexico	*B. rapa ssp. sylvestris*	Spontaneous	WF_MEXICO
Celiabawino1	Mexico	*B. rapa ssp. sylvestris*	Spontaneous	WF_MEXICO
Celiabawino2	Mexico	*B. rapa ssp. sylvestris*	Spontaneous	WF_MEXICO
Celiabawino3	Mexico	*B. rapa ssp. sylvestris*	Spontaneous	WF_MEXICO
Celiabawino4	Mexico	*B. rapa ssp. sylvestris*	Spontaneous	WF_MEXICO

Choguitaavena1	Mexico	*B. rapa ssp. sylvestris*	Spontaneous	WF_MEXICO
Choguitaworkshopb2	Mexico	*B. rapa ssp. sylvestris*	Spontaneous	WF_MEXICO
Choguitaworkshopb4	Mexico	*B. rapa ssp. sylvestris*	Spontaneous	WF_MEXICO
Choguitaworkshopb5	Mexico	*B. rapa ssp. sylvestris*	Spontaneous	WF_MEXICO
Choguitaworkshopb6	Mexico	*B. rapa ssp. sylvestris*	Spontaneous	WF_MEXICO
Claudia1	Mexico	*B. rapa ssp. sylvestris*	Spontaneous	WF_MEXICO
Claudia2	Mexico	*B. rapa ssp. sylvestris*	Spontaneous	WF_MEXICO
Cocham10	Mexico	*B. rapa ssp. sylvestris*	Spontaneous	WF_MEXICO
Cochararehouse1	Mexico	*B. rapa ssp. sylvestris*	Spontaneous	WF_MEXICO
Cochararehouse2	Mexico	*B. rapa ssp. sylvestris*	Spontaneous	WF_MEXICO
Cochararemilpa1	Mexico	*B. rapa ssp. sylvestris*	Spontaneous	WF_MEXICO
Cochararemilpa2	Mexico	*B. rapa ssp. sylvestris*	Spontaneous	WF_MEXICO
Cochararemilpa3	Mexico	*B. rapa ssp. sylvestris*	Spontaneous	WF_MEXICO
Cochararemilpa4	Mexico	*B. rapa ssp. sylvestris*	Spontaneous	WF_MEXICO
Cocheram2	Mexico	*B. rapa ssp. sylvestris*	Spontaneous	WF_MEXICO
Creel3	Mexico	*B. rapa ssp. sylvestris*	Spontaneous	WF_MEXICO
Creel4	Mexico	*B. rapa ssp. sylvestris*	Spontaneous	WF_MEXICO
espinaca10	Mexico	*B. rapa ssp. sylvestris*	Spontaneous	WF_MEXICO
espinaca5	Mexico	*B. rapa ssp. sylvestris*	Spontaneous	WF_MEXICO
espinaca6	Mexico	*B. rapa ssp. sylvestris*	Spontaneous	WF_MEXICO
espinaca7	Mexico	*B. rapa ssp. sylvestris*	Spontaneous	WF_MEXICO
espinaca8	Mexico	*B. rapa ssp. sylvestris*	Spontaneous	WF_MEXICO
espinaca9	Mexico	*B. rapa ssp. sylvestris*	Spontaneous	WF_MEXICO
EspinacaB1	Mexico	*B. rapa ssp. sylvestris*	Spontaneous	WF_MEXICO
EspinacaB2	Mexico	*B. rapa ssp. sylvestris*	Spontaneous	WF_MEXICO
EspinacaB3	Mexico	*B. rapa ssp. sylvestris*	Spontaneous	WF_MEXICO
EspinacaB4	Mexico	*B. rapa ssp. sylvestris*	Spontaneous	WF_MEXICO
InoatfieldRanchoblanco1	Mexico	*B. rapa ssp. sylvestris*	Spontaneous	WF_MEXICO
InoatfieldRanchoblanco2	Mexico	*B. rapa ssp. sylvestris*	Spontaneous	WF_MEXICO
InoatfieldRanchoblanco3	Mexico	*B. rapa ssp. sylvestris*	Spontaneous	WF_MEXICO
InoatfieldRanchoblanco4	Mexico	*B. rapa ssp. sylvestris*	Spontaneous	WF_MEXICO
IntownSanJuanito1	Mexico	*B. rapa ssp. sylvestris*	Spontaneous	WF_MEXICO
Jim1	Mexico	*B. rapa ssp. sylvestris*	Spontaneous	WF_MEXICO
lupeworkshop1	Mexico	*B. rapa ssp. sylvestris*	Spontaneous	WF_MEXICO
Margar1	Mexico	*B. rapa ssp. sylvestris*	Spontaneous	WF_MEXICO
Margar2	Mexico	*B. rapa ssp. sylvestris*	Spontaneous	WF_MEXICO
Mariomilpa1	Mexico	*B. rapa ssp. sylvestris*	Spontaneous	WF_MEXICO
Mariomilpa3	Mexico	*B. rapa ssp. sylvestris*	Spontaneous	WF_MEXICO
Mariomilpa4	Mexico	*B. rapa ssp. sylvestris*	Spontaneous	WF_MEXICO
Mariomilpa6	Mexico	*B. rapa ssp. sylvestris*	Spontaneous	WF_MEXICO
Mariomilpa7	Mexico	*B. rapa ssp. sylvestris*	Spontaneous	WF_MEXICO
Marioseedbankb10	Mexico	*B. rapa ssp. sylvestris*	Spontaneous	WF_MEXICO
Marioseedbankb5	Mexico	*B. rapa ssp. sylvestris*	Spontaneous	WF_MEXICO
Marioseedbankb6	Mexico	*B. rapa ssp. sylvestris*	Spontaneous	WF_MEXICO
Marioseedbankb7	Mexico	*B. rapa ssp. sylvestris*	Spontaneous	WF_MEXICO
Marioseedbankb8	Mexico	*B. rapa ssp. sylvestris*	Spontaneous	WF_MEXICO
Marioseedbankb9	Mexico	*B. rapa ssp. sylvestris*	Spontaneous	WF_MEXICO
Mariosembrado1	Mexico	*B. rapa ssp. sylvestris*	Spontaneous	WF_MEXICO
Mariosembrado2	Mexico	*B. rapa ssp. sylvestris*	Spontaneous	WF_MEXICO
Mariosembrado3	Mexico	*B. rapa ssp. sylvestris*	Spontaneous	WF_MEXICO
Mariosembrado4	Mexico	*B. rapa ssp. sylvestris*	Spontaneous	WF_MEXICO
Nacho20071	Mexico	*B. rapa ssp. sylvestris*	Spontaneous	WF_MEXICO
Nacho20072	Mexico	*B. rapa ssp. sylvestris*	Spontaneous	WF_MEXICO
Nacho20073	Mexico	*B. rapa ssp. sylvestris*	Spontaneous	WF_MEXICO
Nacho20074	Mexico	*B. rapa ssp. sylvestris*	Spontaneous	WF_MEXICO
Nacho2007b5	Mexico	*B. rapa ssp. sylvestris*	Spontaneous	WF_MEXICO
Nacho2007b6	Mexico	*B. rapa ssp. sylvestris*	Spontaneous	WF_MEXICO
Nacho2007b7	Mexico	*B. rapa ssp. sylvestris*	Spontaneous	WF_MEXICO
Nacho2007b8	Mexico	*B. rapa ssp. sylvestris*	Spontaneous	WF_MEXICO
NGB1320671Branbjerg	Mexico	*B. rapa ssp. sylvestris*	Spontaneous	WF_MEXICO

Ozumbacriollo1	Mexico	*B. rapa ssp. sylvestris*	Spontaneous	WF_MEXICO
Ranchoblanco13	Mexico	*B. rapa ssp. sylvestris*	Spontaneous	WF_MEXICO
Ranchoblanco2	Mexico	*B. rapa ssp. sylvestris*	Spontaneous	WF_MEXICO
Ranchoblanco5	Mexico	*B. rapa ssp. sylvestris*	Spontaneous	WF_MEXICO
Ranchoblanco6	Mexico	*B. rapa ssp. sylvestris*	Spontaneous	WF_MEXICO
Ranchoblanco7	Mexico	*B. rapa ssp. sylvestris*	Spontaneous	WF_MEXICO
Ranchoblanco9	Mexico	*B. rapa ssp. sylvestris*	Spontaneous	WF_MEXICO
Refu1	Mexico	*B. rapa ssp. sylvestris*	Spontaneous	WF_MEXICO
Refu11	Mexico	*B. rapa ssp. sylvestris*	Spontaneous	WF_MEXICO
Refu12	Mexico	*B. rapa ssp. sylvestris*	Spontaneous	WF_MEXICO
Refu2	Mexico	*B. rapa ssp. sylvestris*	Spontaneous	WF_MEXICO
Refu25	Mexico	*B. rapa ssp. sylvestris*	Spontaneous	WF_MEXICO
Refu3	Mexico	*B. rapa ssp. sylvestris*	Spontaneous	WF_MEXICO
Refu4	Mexico	*B. rapa ssp. sylvestris*	Spontaneous	WF_MEXICO
Refu5	Mexico	*B. rapa ssp. sylvestris*	Spontaneous	WF_MEXICO
Refu6	Mexico	*B. rapa ssp. sylvestris*	Spontaneous	WF_MEXICO
Refu7	Mexico	*B. rapa ssp. sylvestris*	Spontaneous	WF_MEXICO
Refu8	Mexico	*B. rapa ssp. sylvestris*	Spontaneous	WF_MEXICO
Refu9	Mexico	*B. rapa ssp. sylvestris*	Spontaneous	WF_MEXICO
Refugio2	Mexico	*B. rapa ssp. sylvestris*	Spontaneous	WF_MEXICO
Refugio3	Mexico	*B. rapa ssp. sylvestris*	Spontaneous	WF_MEXICO
Rejo1	Mexico	*B. rapa ssp. sylvestris*	Spontaneous	WF_MEXICO
Rejo2	Mexico	*B. rapa ssp. sylvestris*	Spontaneous	WF_MEXICO
Rejo3	Mexico	*B. rapa ssp. sylvestris*	Spontaneous	WF_MEXICO
Rejo4	Mexico	*B. rapa ssp. sylvestris*	Spontaneous	WF_MEXICO
Rejogochib10	Mexico	*B. rapa ssp. sylvestris*	Spontaneous	WF_MEXICO
Rejogochib5	Mexico	*B. rapa ssp. sylvestris*	Spontaneous	WF_MEXICO
Rejogochib6	Mexico	*B. rapa ssp. sylvestris*	Spontaneous	WF_MEXICO
Rejogochib7	Mexico	*B. rapa ssp. sylvestris*	Spontaneous	WF_MEXICO
Rejogochib8	Mexico	*B. rapa ssp. sylvestris*	Spontaneous	WF_MEXICO
Sanignacmb10	Mexico	*B. rapa ssp. sylvestris*	Spontaneous	WF_MEXICO
Sanignacmb6	Mexico	*B. rapa ssp. sylvestris*	Spontaneous	WF_MEXICO
Sanignacmb7	Mexico	*B. rapa ssp. sylvestris*	Spontaneous	WF_MEXICO
Sanignacmb8	Mexico	*B. rapa ssp. sylvestris*	Spontaneous	WF_MEXICO
Sanignacmb9	Mexico	*B. rapa ssp. sylvestris*	Spontaneous	WF_MEXICO
Santiag1	Mexico	*B. rapa ssp. sylvestris*	Spontaneous	WF_MEXICO
Santiag2	Mexico	*B. rapa ssp. sylvestris*	Spontaneous	WF_MEXICO
Sisoguichi1	Mexico	*B. rapa ssp. sylvestris*	Spontaneous	WF_MEXICO
Vainooo1	Mexico	*B. rapa ssp. sylvestris*	Spontaneous	WF_MEXICO
Vainooo2	Mexico	*B. rapa ssp. sylvestris*	Spontaneous	WF_MEXICO
Vainooo3	Mexico	*B. rapa ssp. sylvestris*	Spontaneous	WF_MEXICO
BRA2218Canada	Canada	*B. rapa ssp. sylvestris*	Spontaneous	WF_NORTH_AMERICA
Quebecemilyharvest	Canada	*B. rapa ssp. sylvestris*	Spontaneous	WF_NORTH_AMERICA
Quebecsimard2	Canada	*B. rapa ssp. sylvestris*	Spontaneous	WF_NORTH_AMERICA
acm05	U.S.	*Brassica rapa*	wild/feral	WF_NORTH_AMERICA
Ames 30080	U.S.	*Brassica rapa*		WF_NORTH_AMERICA
Ames 30083	U.S.	*Brassica rapa*		WF_NORTH_AMERICA
Ames 30084	U.S.	*Brassica rapa*		WF_NORTH_AMERICA
Arg1	Argentina	*B. rapa ssp. sylvestris*	Spontaneous	WF_SOUTH_AMERICA
Arg2	Argentina	*B. rapa ssp. sylvestris*	Spontaneous	WF_SOUTH_AMERICA
311711Chile	Chile	*B. rapa ssp. sylvestris*	Spontaneous	WF_SOUTH_AMERICA
CR2300Colom	Colombia	*B. rapa ssp. sylvestris*	Spontaneous	WF_SOUTH_AMERICA
Ecualf2	Ecuador	*B. rapa ssp. sylvestris*	Spontaneous	WF_SOUTH_AMERICA
Ecualf3	Ecuador	*B. rapa ssp. sylvestris*	Spontaneous	WF_SOUTH_AMERICA
GuatUSDA1	Guatemala	*B. rapa ssp. sylvestris*	Spontaneous	WF_SOUTH_AMERICA
GuatUSDA2	Guatemala	*B. rapa ssp. sylvestris*	Spontaneous	WF_SOUTH_AMERICA
Bol1	Latin America	*B. rapa ssp. sylvestris*	Spontaneous	WF_SOUTH_AMERICA
Bol2	Latin America	*B. rapa ssp. sylvestris*	Spontaneous	WF_SOUTH_AMERICA
bolquestion	Latin America	*B. rapa ssp. sylvestris*	Spontaneous	WF_SOUTH_AMERICA
CR2299colo	Latin America	*B. rapa ssp. sylvestris*	Spontaneous	WF_SOUTH_AMERICA

Lamay	Latin America	*B. rapa ssp. sylvestris*	Spontaneous	WF_SOUTH_AMERICA
Panalf1	Latin America	*B. rapa ssp. sylvestris*	Spontaneous	WF_SOUTH_AMERICA
Panalf5	Latin America	*B. rapa ssp. sylvestris*	Spontaneous	WF_SOUTH_AMERICA
Panalfg4	Latin America	*B. rapa ssp. sylvestris*	Spontaneous	WF_SOUTH_AMERICA
Peru1	Peru	*B. rapa ssp. sylvestris*	Spontaneous	WF_SOUTH_AMERICA
PI 162778	Argentina	*Brassica rapa*		WF_SOUTH_AMERICA
PI 633181	Honduras	*B.rapa subsp.rapa*		WF_SOUTH_AMERICA
PI 217931	Pakistan	*Brassica rapa*	Sarson	YS
PI 164841	India	*Brassica rapa*	yellow sarson	YS
PI 165608	India	*Brassica rapa*	yellow sarson	YS
PI 175052	India	*Brassica rapa*	yellow sarson	YS
PI 346882	India	*B.rapa subsp.trilocularis*	yellow sarson	YS
PI 347608	India	*B.rapa subsp.trilocularis*	yellow sarson	YS
PI 352811	India	*Brassica rapa*	yellow sarson	YS
PI 352822	India	*Brassica rapa*	yellow sarson	YS
PI 352825	India	*Brassica rapa*	yellow sarson	YS
PI 370737	India	*Brassica rapa*	yellow sarson	YS
PI 459016	India	*B.rapa subsp.trilocularis*	yellow sarson	YS
PI 459017	India	*B.rapa subsp.trilocularis*	yellow sarson	YS
PI 459019	India	*B.rapa subsp.trilocularis*	yellow sarson	YS
PI 459020	India	*B.rapa subsp.trilocularis*	yellow sarson	YS
PI 459021	India	*B.rapa subsp.trilocularis*	yellow sarson	YS
PI 459023	India	*B.rapa subsp.trilocularis*	yellow sarson	YS
PI 603020	India	*Brassica rapa*	yellow sarson	YS
PI 649160	India	*Brassica rapa*	yellow sarson	YS
PI 649198	India	*B.rapa subsp.trilocularis*	yellow sarson	YS
PI 649199	India	*B.rapa subsp.trilocularis*	yellow sarson	YS
PI 649200	India	*B.rapa subsp.trilocularis*	yellow sarson	YS
PI 649201	India	*B.rapa subsp.trilocularis*	yellow sarson	YS
PI 649202	India	*B.rapa subsp.trilocularis*	yellow sarson	YS
PI 390142	Pakistan	*B.rapa subsp.trilocularis*	yellow sarson	YS
PI 426420	Pakistan	*B.rapa subsp.trilocularis*	yellow sarson	YS
PI 426422	Pakistan	*B.rapa subsp.trilocularis*	yellow sarson	YS
PI 426423	Pakistan	*B.rapa subsp.trilocularis*	yellow sarson	YS
PI 649164	Pakistan	*Brassica rapa*	yellow sarson	YS
PI 649204	U.S.	*B.rapa subsp.trilocularis*	yellow sarson	YS
PI 649206	U.S.	*B.rapa subsp.trilocularis*	yellow sarson	YS
PI 649207	U.S.	*B.rapa subsp.trilocularis*	yellow sarson	YS
PI 649208	U.S.	*B.rapa subsp.trilocularis*	yellow sarson	YS

Table S2. List of *Brassica rapa* accessions used in this study, including their scientific names and classification into population groups based on geographic origin and cultivated type.

Scientific name	Region	Morphotype	Group
B. rapa ssp. *sylvestris* var. *esculenta*	Europe	Leafy	Grelos
B. rapa ssp. *sylvestris* var. *esculenta*	Europe	Leafy	Rapini
Brassica rapa subsp. *chinensis*	Asia	Leafy	Bok choy Asia
Brassica rapa subsp. *dichotoma*	Asia	Oilseed	Toria Asia
Brassica rapa subsp. *dichotoma*	Asia	Oilseed	Brown sarson Asia
Brassica rapa subsp. *narinosa*	Asia	Leafy	Tatsoi Asia
Brassica rapa subsp. *nipposinica*	Asia	Leafy	Mizuna Asia
Brassica rapa subsp. *oleifera*	Asia	Oilseed	Oilseed rape Asia
Brassica rapa subsp. *oleifera*	Europe	Oilseed	Oilseed rape Europe
Brassica rapa subsp. *parachinensis*	Asia	Leafy	Choy sum Asia
Brassica rapa subsp. *pekinensis*	Asia	Leafy	Napa cabbage Asia
Brassica rapa subsp. *pekinensis*	North America	Leafy	Napa cabbage North America
Brassica rapa subsp. *perviridis*	North America	Leafy	Komatsuna North America
Brassica rapa subsp. r*apa*	Asia	Turnip	Turnip Asia
Brassica rapa subsp. *rapa*	North America	Turnip	Turnip North America
Brassica rapa subsp. *rapa*	Europe	Turnip	Turnip Europe
Brassica rapa subsp. *rapa*	Africa	Turnip	Turnip Africa
Brassica rapa subsp. *sylvestris*	Caucasus	Wild	Wild Caucasus
Brassica rapa subsp. *sylvestris*	North America	Weedy	Weedy North America
Brassica rapa subsp. *sylvestris*	South America	Weedy	Weedy South America
Brassica rapa subsp. *sylvestris*	Europe	Weedy	Weedy Europe
Brassica rapa subsp. *sylvestris*	Africa	Weedy	Weedy Africa
Brassica rapa subsp. *sylvestris*	Asia	Weedy	Weedy Asia
Brassica rapa subsp. *sylvestris*	Mexico	Weedy	Weedy Mexico
Brassica rapa subsp. *sylvestris*	Argentina	Weedy	Weedy Argentina
Brassica rapa subsp. *trilocularis*	Asia	Oilseed	Yellow Sarson

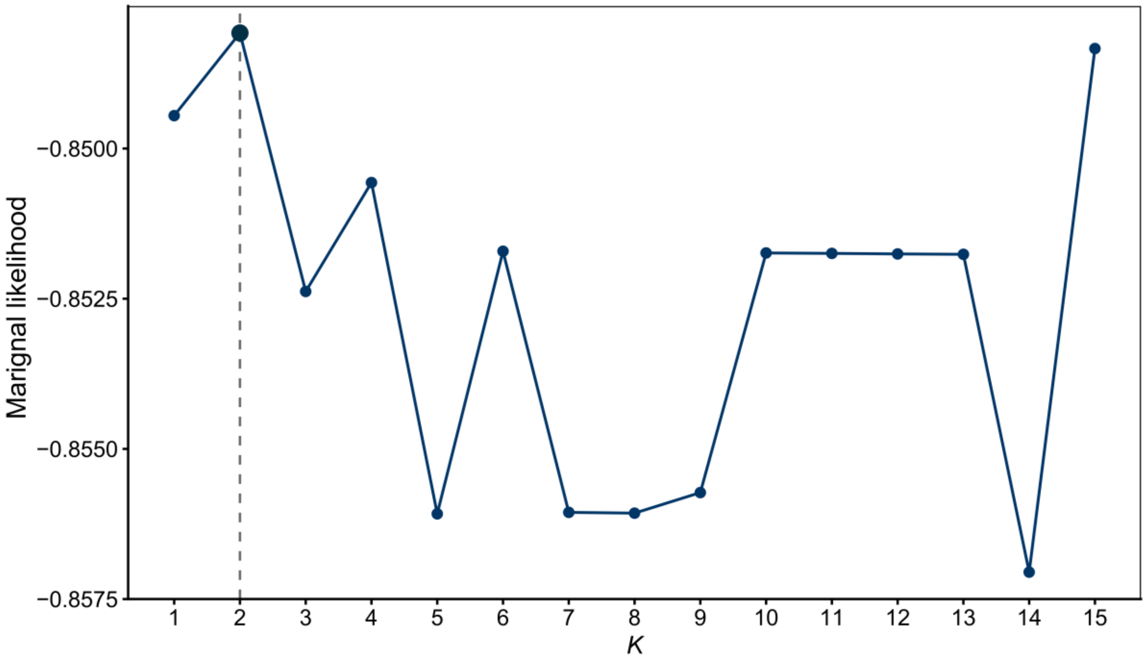

Figure S1. Variational marginal likelihood across K values used to determine the optimal number of genetic clusters (K) among Argentinian accessions.

Table S3. Variational marginal likelihood across K values among Argentinian accessions, inferred by fastStructure. The highest value was observed at K = 2, which was selected as the optimal number of genetic clusters.

K	Marginal Likelihood
1	-0.8494529460
2	-0.8480770594
3	-0.8523794621
4	-0.8505667948
5	-0.8560807960
6	-0.8517100778
7	-0.8560565571
8	-0.8560698461
9	-0.8557271340
10	-0.8517383653
11	-0.8517456643
12	-0.8517547843
13	-0.8517610973
14	-0.8570515215
15	-0.8483358108

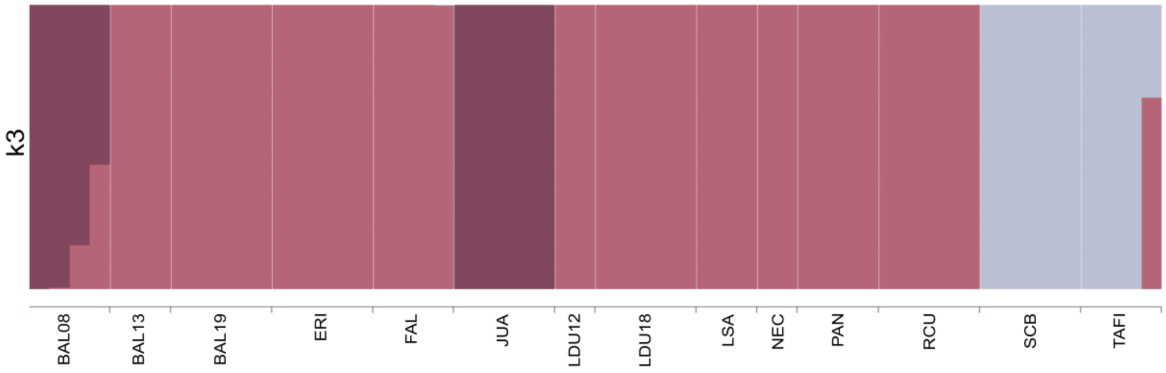

Figure S2. FastStructure plot showing the population structure of Argentinian *Brassica rapa* accessions at K = 3.

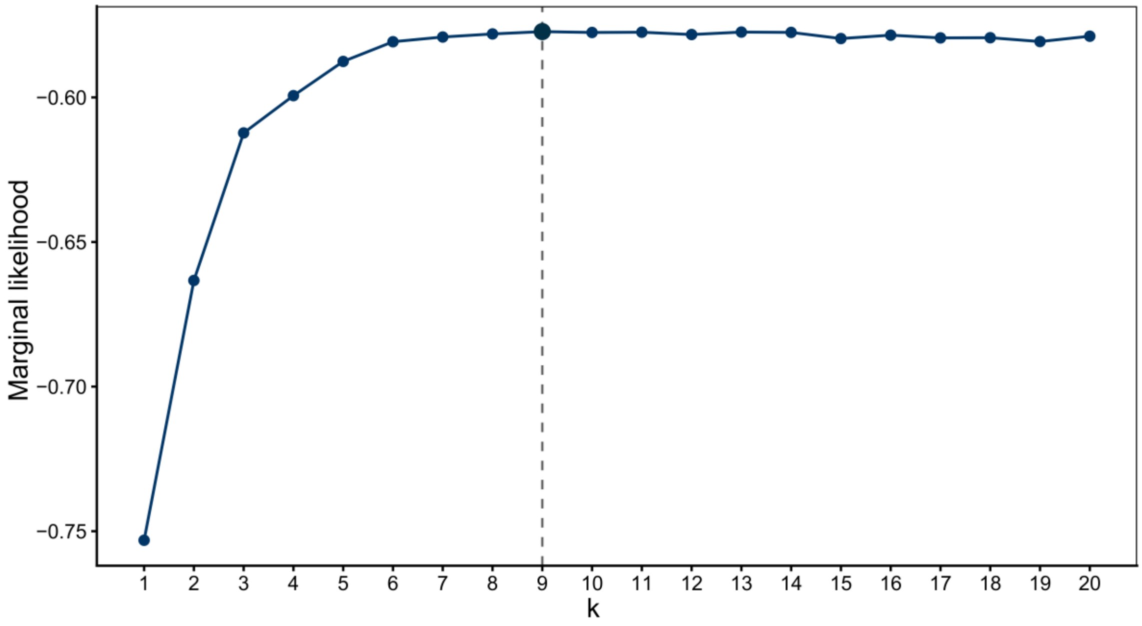

Figure S3. Variational marginal likelihood across K values used to determine the optimal number of genetic clusters (K).

Table S4. Variational marginal likelihood across K values inferred by fastStructure. The highest value was observed at K = 9, which was selected as the optimal number of genetic clusters.

k	***Marginal likelihood***
1	-0.753139128
2	-0.663300535
3	-0.612289245
4	-0.599396511
5	-0.587563818
6	-0.580747761
7	-0.579113407
8	-0.578052927
9	-0.57724183
10	-0.577548202
11	-0.57745800
12	-0.578261206
13	-0.577408992
14	-0.577522722
15	-0.579648188
16	-0.578478927
17	-0.579421417
18	-0.57935635
19	-0.580692441
20	-0.578839263

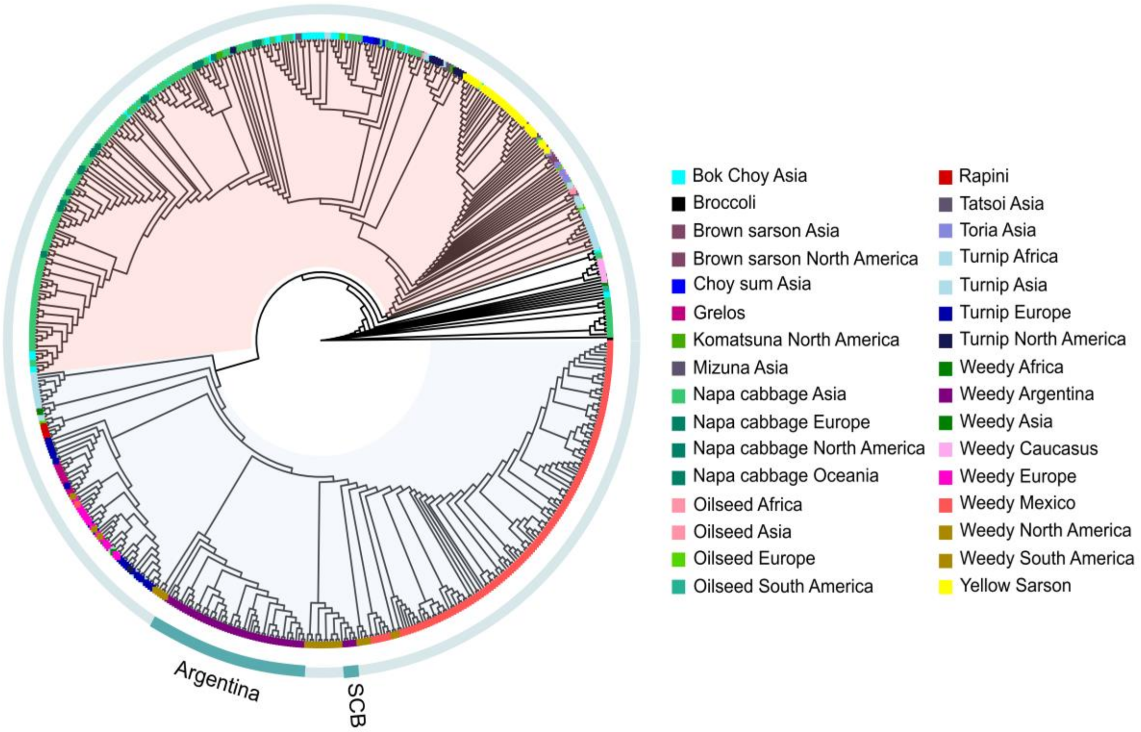

Figure S4. Neighbor-joining tree showing the relationships between Argentinean *B. rapa* accessions, crops, and spontaneous accessions from worldwide populations. Different colors represent distinct biotypes and sample regions.

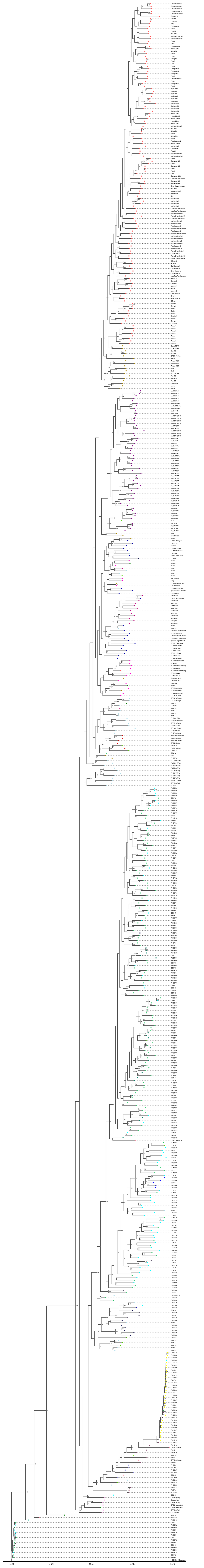

Figure S5. Maximum likelihood tree (RAxML) of 624 *Brassica rapa* individuals, including 56 Argentinian samples and 568 accessions from around the world. Boostrap support values are shown next to nodes.
